# *Ab Initio* and Statistical Rate Theory Exploration of the CH (X^2^Π) + OCS Gas-Phase Reaction

**DOI:** 10.1021/acs.jpca.3c01082

**Published:** 2023-07-28

**Authors:** Daniel I. Lucas, Casey J. Kavaliauskas, Mark A. Blitz, Dwayne E. Heard, Julia H. Lehman

**Affiliations:** † School of Chemistry, University of Birmingham, Edgbaston B15 2TT, United Kingdom; ‡ School of Chemistry, University of Leeds, Leeds LS2 9JT, United Kingdom; § National Centre for Atmospheric Science, University of Leeds, Leeds LS2 9JT, United Kingdom

## Abstract

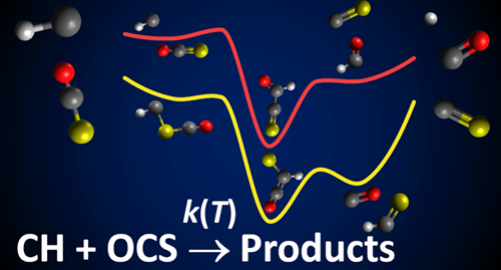

The first theoretical results regarding the gas-phase reaction mechanism and kinetics of the CH (X^2^Π) + OCS reaction are presented here. This reaction has a proposed importance in the removal of OCS in regions of the interstellar medium (ISM) and has the potential to form the recently observed HCS/HSC isomers, with both constitutional isomers having recently been observed in the L483 molecular cloud in a 40:1 ratio. Statistical rate theory simulations were performed on stationary points along the reaction potential energy surface (PES) obtained from *ab initio* calculations at the RO-CCSD(T)/aug-cc-pV(Q+d)Z//M06-2X-D3/aug-cc-pV(Q+d)Z level of theory over the temperature and total density range of 150–3000 K and 10^11^–10^24^ cm^–3^, respectively, using a Master Equation analysis. Exploration of the reaction potential energy surface revealed that all three pathways identified to create CS + HCO products required surmounting barriers of 16.5 kJ mol^–1^ or larger when CH approached the oxygen side of OCS, rendering this product formation negligible below 1000 K, and certainly under low-temperature ISM conditions. In contrast, when CH approaches the sulfur side of OCS, only submerged barriers are found along the reaction potential energy surface to create HCCO + S or CO + HCS, both of which are formed via a strongly bound OCC(H)S intermediate (−358.9 kJ mol^–1^). Conversion from HCS to HSC is possible *via* a barrier of 77.8 kJ mol^–1^, which is still −34.1 kJ mol^–1^ below the CH + OCS entrance channel. No direct route from CH + OCS to H + CO + CS was found from our *ab initio* calculations. Rate theory simulations suggest that the reaction has a strong negative temperature dependence, in accordance with the barrierless addition of CH to the sulfur side of OCS. Product branching fractions were also determined from MESMER simulations over the same temperature and total density range. The product branching fraction of CO + HCS reduces from 79% at 150 K to 0.0% at 800 K, while that of HCS dissociation to H + CS + CO increases from 22% at 150 K to 100% at 800 K. The finding of CO + HCS as the major product at the low temperatures relevant to the ISM, instead of H + CS + CO, is in opposition to the current supposition used in the KIDA database and should be adapted in astrochemical models as another source of the HCS isomer.

## Introduction

The chemical reactivities and abundances of sulfur and sulfur-containing molecules provide an intriguing challenge to researchers in the fields of astrochemistry, atmospheric chemistry, and the study of planetary atmospheres. Relative abundances of small sulfur-containing molecules and their isotope fractionation can be used to provide insight into the physical properties of star-forming regions of space^1−9^ and to follow the evolution of hot cores.^10−12^ More recently, Li et al. suggested that the HSO and HOSO radicals may play a key role in HCO and HOCO radical production through photo-induced hydrogen atom transfer reactions of sulfur-containing molecules in CO and CO_2_ ices.^13^ An example of a small but important sulfur-bearing species is carbonyl sulfide (OCS).^14,15^ For example, OCS is the most abundant sulfur-containing molecule in Earth’s atmosphere, with a mixing ratio of ∼500 ppt.^16^ Moreover, OCS contributes to stratospheric sulfate aerosol formation, due to the long tropospheric lifetime of OCS (τ > 2 years) allowing transportation to the stratosphere.^17−19^ In the Venusian atmosphere, a rapid reduction in the OCS mixing ratio from 10 to 20 ppm at an altitude of 30 km, to 0.35 ppm at 38 km, is observed, with reasons for this decline still unknown.^20,21^ Furthermore, OCS was found to exist in several Jupiter family comets^22^ and, more recently, in the L1455-IRS1 and L1551-IRS5 class I protostars.^23^

One astrophysical environment of chemical interest is the interstellar medium (ISM), the region of space between stars and planets in a galaxy. The methylidyne radical, CH,^24^ carbonyl sulfide, OCS,^25^ and a selection of other small sulfur-containing molecules^26^ are among the ∼270 molecules detected in interstellar and circumstellar environments of space.^14^ Despite this, astrochemical models still fail to accurately capture the chemistry of sulfur-containing molecular species due to the phenomenon of sulfur depletion. In brief, dark cloud astrochemical models that incorporate observed abundances of sulfur-bearing molecules estimate a cosmic abundance of gas-phase atomic sulfur that is severely depleted by up to ∼2 orders of magnitude from the observed value of ∼10^–5^ relative to the abundance of H.^27−35^ Similar trends have also been observed for astrochemical models of hot cores, hot corinos, and bipolar outflows.^35^ Several improved astrochemical models have attempted to provide insight into sulfur depletion, predicting that the bulk of sulfur resides in the form of atomic S, solid H_2_S or OCS,^31,33,36,37^ and in S_4_ and S_8_ allotropic forms, among others.^34^ However, the gas-phase abundances of organosulfur molecules are still not accurately described by astrochemical models.^31,35^ This is evident in a recent dense molecular cloud model in which the relative abundance of the thioformyl radical, HCS, the ratio of H_2_CS:HCS, and the observation of the metastable iso-thioformyl radical, HSC, could not match values from astronomical observations. The observed fractional abundances of HCS and HSC were 10^–10^ and 10^–12^ relative to H_2_, respectively, and the H_2_CS:HCS ratio was found to be ∼1.^38^ However, the astrochemical model of Vidal et al. predicts an HCS abundance of at least 1 order of magnitude lower than that observed and a H_2_CS abundance that is 1–2 orders of magnitude greater than HCS.^31^ Furthermore, another recent protoplanetary disk model underpredicts the abundance of H_2_CS by 1–2 orders of magnitude compared to astronomical observations.^39^ Therefore, significant advances in the understanding of sulfur chemistry are required in order to improve model predictions.

Astrochemical models employ a very large chemical reaction network that includes reaction rate coefficients and branching ratios of gas-phase chemical reactions. In models of sulfur-containing molecules, organooxygen kinetic data are often used in place of the missing organosulfur data due to a lack of data on the molecular reactivity of organosulfur species.^40^ Sulfur and oxygen are valence isoelectronic, suggesting this is a reasonable approximation at first glance. However, the reactivity of sulfur- and oxygen-containing molecular species can be very different. For example, the SH and OH radicals show similar reactivity in a long-range radical–radical recombination reaction with NO_2_, whereas the SH radical is found to react at least 3 orders of magnitude slower than the OH radical in the radical–molecule reaction with C_2_H_4_ at room temperature.^41−44^ The lack of data pertaining to the chemical reactivity of sulfur-containing molecules impacts the reliability of models of astrophysical environments of interest and the model’s ability to reproduce the observed molecular abundances of sulfur-containing molecules.

The CH + OCS reaction has been suggested to be an important sink of gas-phase OCS in cold astrophysical environments,^45^ and also has the potential to produce the recently observed thioformyl, HCS, and iso-thioformyl, HSC, radical isomers.^38^ However, to the best of our knowledge, there exists only one experimental investigation over the temperature range of 297–667 K for the title reaction.^46^ In this flow cell experiment, the pulsed laser photolysis—laser-induced fluorescence technique—was used to follow the decay in the CH radical in the presence of a known excess OCS concentration to derive the temperature-dependent reaction rate coefficients, *k*(*T*). The authors reported a slight decrease in the value of *k*(*T*) with increasing temperature from *k*(297 K) = 3.9 × 10^–10^ cm^3^ s^–1^ to *k*(667 K) = 2.7 × 10^–10^ cm^3^ s^–1^ and did not report the identity of any reaction products. However, extrapolation of the experimental Arrhenius expression to low temperatures results in unrealistic values of the reaction rate coefficient, with *k*(30 K) = 1.1 × 10^–7^ cm^3^ s^–1^. Consequently, Loison et al. recommend that an upper limit of 4.0 × 10^–10^ cm^3^ s^–1^ to the value of *k*(*T*) at 300 K should be employed in astrochemical models over the temperature range of 10–300 K.^45^ Additionally, the negative temperature dependence observed coupled with the relatively large value of the temperature-dependent reaction rate coefficient suggests the possibility of a reaction that is barrierless in nature.

While no prior theoretical investigations on the title reaction have been reported in the literature, one comparable system that has been studied theoretically is that of the ground-state CH radical with CO_2_.^47^ In this work, *ab initio* calculations performed at the CCSD(T)/CBS//CCSD/cc-pVDZ level of theory found that CH inserts into one of the C–O bonds of CO_2_, and then undergoes either a further two-step or three-step reaction mechanism. Subsequent transition state theory calculations then found that the formation of CO + HCO dominates below 300 K, while H + 2CO formation is preferred above 300 K. A comparison between the valence isoelectronic CH + OCS and CH + CO_2_ systems is one valuable way to begin to elucidate differences in the reaction mechanism of organooxygen and organosulfur species.

Analogous to the CH + CO_2_ reaction forming CO + HCO, the reaction of CH + OCS presents an opportunity for the formation of CO + HCS, and particularly for exploring the relative importance of the constitutional isomers HCS and HSC in this reaction. The isomerization and decomposition of the thioformyl radical have been studied previously using computational methods.^48−51^ Yamada et al.^51^ studied the doublet HCS PES through the association of CH + S or SH + C at the CCSD(T)/aug-cc-pVTZ//B3LYP/6-311G(d,p) level of theory, reporting that HCS is 159 kJ mol^–1^ more stable than HSC and a 77 kJ mol^–1^ barrier to isomerization *via* a cyclic transition state. This agrees with the studies by Puzzarini,^50^ on the HCS/HSC and HCS^+^/HSC^+^ isomers at the (R)CCSD(T) level of theory with complete basis set extrapolation, and Galland et al.,^48^ on the gas-phase reaction of C + H_2_S at the CCSD(T)/cc-pVTZ//QCISD/cc-pVDZ level of theory, who report barriers of 84 and 76 kJ mol^–1^, respectively. Furthermore, Yamada et al.^51^ found that HCS decomposes in a barrierless process, whereas HSC decomposes to H + CS via a 15 kJ mol^–1^ exit barrier with respect to the decomposition products. This contrasts with the study by Galland et al.^48^ who report a 5 kJ mol^–1^ exit barrier to HCS decomposition and a 20 kJ mol^–1^ exit barrier to HSC decomposition, relative to the decomposition products. Moreover, Galland et al.^48^ were not able to determine the relative HCS:HSC branching ratio experimentally and found only a 0.05% contribution of the total reaction rate coefficient in forming HSC + H from their rate theory simulations. Currently, the kinetic database for astrochemistry (KIDA) also recommends based on the work of Loison et al.^45^ that the sole products of the CH + OCS reaction are H + CS + CO over all temperatures. The lack of experimental and theoretical data for the CH + OCS system, coupled with issues surrounding sulfur depletion in the ISM, prompted us to investigate the reaction mechanism of CH + OCS further. Based on available enthalpies of formation at 298 K for reactant and possible product species from experiment,^52−54^ calculation,^50^ or the NIST-JANAF thermochemical tables,^55^ the possible exothermic product channels for the CH + OCS reaction are
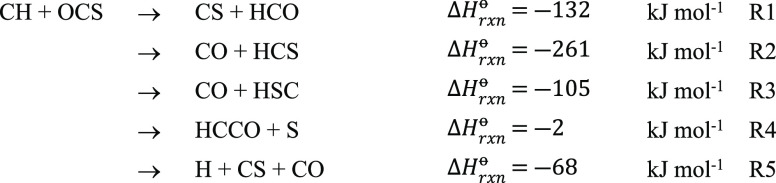
In this paper, we present the first theoretical investigation for the gas-phase neutral–neutral reaction of the CH (X^2^Π) radical with OCS, using a combination of *ab initio* calculations and statistical rate theory simulations. Predicted rate coefficients for the title reaction are also reported over the temperature and total density ranges of 150–3000 K and 10^11^–10^24^ cm^–3^, respectively. The principal aim of the work presented here is to elucidate the reaction mechanism, calculate reaction rate coefficients, and determine product branching fractions for the reaction of interest. Furthermore, comparing this system to that of the CH + CO_2_ reaction provides an opportunity to understand the fundamental differences in chemical reactivity between organosulfur molecules and their oxygenated counterparts.

## Methods

### Electronic Structure Calculations

Geometry optimizations and harmonic frequency calculations presented in this work were performed using the M06-2X-D3 functional^56^ in conjunction with the correlation consistent aug-cc-pV(Q+d)Z Dunning basis set with the Gaussian 09 software package.^57^ Subsequent single point energy calculations on stationary points along the reaction PES were then calculated at the RO-CCSD(T)/aug-cc-pV(Q+d)Z level of theory.

Stationary point structures (reactants, products, intermediates, and transition states) along the potential energy surface (PES) were first optimized before subsequent harmonic vibrational frequency calculations were performed on the minimum energy structures. Minima along the PES were identified as those having all real harmonic vibrational frequencies, while transition state species were identified as those possessing a single imaginary frequency. A vibrational frequency scaling factor of 0.972 was used for the M06-2X-D3/aug-cc-pV(Q+d)Z level of theory.^56^ To verify that the identified transition state species are saddle points that connect two minima via a minimum energy pathway along the PES, intrinsic reaction coordinate (IRC) calculations were performed. In addition, further exploration of the reaction PES was carried out by performing relaxed scans of the entrance channels as CH approaches OCS.

The choice of a hybrid DFT method and the aug-cc-pV(Q+d)Z basis set for geometry and frequency calculation is based on previous work with sulfur-based systems.^58,59^ Other levels of theory were also explored to calculate stationary point structures (reactants, products, and reaction intermediates), as reported in the Supporting Information, but with little change in relative energies or geometries. Geometries, energies, and vibrational frequencies for all stationary points and transition states reported here can be found in the Supporting Information.

### Statistical Rate Theory Calculations

The stationary points on the reaction PES for the CH + OCS reaction were utilized by the Master Equation Solver for Multi Energy-well Reactions (MESMER) software.^60^ MESMER uses the electronic energies, rotational constants, and vibrational frequencies obtained from *ab initio* calculations of the stationary points to determine the total rovibrational energy contained within the system. A one-dimensional energy grained master equation (EGME) is then employed, in which the total rovibrational energy states for each stationary point structure along the reaction PES are divided into energy grains that couples the reactant, intermediate, and product species to each other *via* the microcanonical rate coefficients, *k*(*E*). MESMER considers a series of individual reaction steps, not solely the overall reaction of CH + OCS → products, for example. Hence, for a reaction step with a defined transition state, Rice, Ramsperger, Kassel, and Markus (RRKM) theory was used to calculate *k*(*E*),^61^ whereas an inverse Laplace transform (ILT) method was used for any reaction steps that do not possess a defined transition state.^62^ Implementation of the ILT method in MESMER uses a modified Arrhenius equation taking the form of eq 1
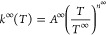
1Activation or deactivation of species between energy grains *via* collisional energy transfer with a third body (for example, N_2_, Ar, or He) is also accounted for in an “exponential down” model described by eq 2. Here, the average energy transferred during a collision, ⟨Δ*E*⟩_d_, is given by a reference value, ⟨Δ*E*⟩_d,ref_, multiplied by the ratio of the temperature of the simulation, *T*, to the reference temperature, *T*_ref_, which is the same as *T*^∞^ in eq 1, raised to a power, *n*
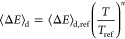
2The typical collisional energy transfer parameters used for MESMER simulations are given in Table S7, and those used specifically in this work are outlined in the text below.

Each individual energy grain is accounted for by a set of coupled differential equations within the one-dimensional EGME model, defined by eq 3, where *p* is the population density vector that contains individual grain populations from each minimum along the PES, and **M** is the transition matrix that describes the temporal change in grain population due to reactive processes and collisional energy transfer
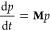
3

Solving the differential equation (eq 3) then gives the population density vector, *p*, represented by eq 4
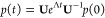
4Here, **U** is a matrix of eigenvectors calculated from diagonalization of the transition matrix, **M**, Λ is the matrix of corresponding eigenvalues, and *p*(0) is the initial population density vector of the energy grains. The smallest eigenvalues contained within Λ are the chemically significant eigenvalues (CSE) from which the Bartis–Widom phenomenological rate coefficients are derived.^63^ Here, the requirement is that the CSEs are much smaller than the internal energy relaxation eigenvalues (IERE).

In this work, temperature and pressure-dependent reaction rate coefficients and product branching fractions were calculated using the MESMER software package.^60^ A summary of the simulation conditions is provided in Table 1. Simulations were performed over a temperature and total density range of 150–3000 K and 10^11^–10^24^ cm^–3^, respectively. This temperature and density range was chosen as it addresses most conditions across the ISM, Earth’s atmosphere, and that used in combustion chemistry. Furthermore, the density range chosen here allows for the examination of the pressure dependence of the title reaction between a low-pressure and a high-pressure limit.

**Table 1:** Summary of MESMER Simulation Conditions Used in This Work

temperature range, K	pressure range, cm^–3^	reaction pathways included	precision	grain size, cm^–1^	ILT parameters
150–300	10^11^–10^24^	P2, P3b	qd	100	*A*^∞^: 4.08 × 10^–10^
					*n*^∞^: −0.03
400–1000	10^11^–10^24^	P1c, P2, P3a, P3b	qd	100	*A*^∞^: 4.08 × 10^–10^
					*n*^∞^: −0.03
1000–3000	10^11^–10^24^	All	dd	200	*A*^∞^: 4.08 × 10^–10^
					*n*^∞^: −0.03

To calculate reaction rate coefficients, MESMER requires a machine-precision value for the calculation and a value for the grain size, i.e., the separation of individual energy grains. Machine-precision values range from double (d), double-double (dd), to quad-double (qd). While it is desirable to have the smallest possible grain size and highest possible machine precision, a balance between computational cost and the accuracy of the simulations must be achieved. Generally, the specified grain size should always be smaller than the collisional energy transfer value at the temperature of the simulation. This was kept in mind when choosing the machine-precision and grain size values outlined in Table 1. In addition to reaction rate coefficients, product branching fractions as a function of reaction time can also be calculated from time-dependent concentrations of each species involved in the reaction. However, at low temperatures when a very low-energy intermediate is present along the reaction PES, numerical difficulty arises when running simulations at the highest-precision (qd) arithmetic to solve the EGME, as experienced in previous use of MESMER simulations.^64^ Due to the presence of a very low-energy intermediate along the reaction PES (P2INT2, as discussed below), simulations below 150 K for the overall reaction rate coefficient were not possible.^64^ For these reasons, simulations over the temperature range of 1000–3000 K were performed on the entire reaction PES, while simulations over the temperature range of 300–1000 K were performed on reaction pathways P1c, P2, P3a, and P3b, only. Simulations at 1000 K were repeated at two different machine-precision and grain size values to ensure changing the accuracy of the simulation did not alter the results. Simulations over the temperature range of 150–300 K excluded all reaction pathways except P2 and P3b.

To accommodate for collisional energy transfer, an N_2_ bath gas was used for all simulations, with values of 250 cm^–1^, 298 K, and 0.25 used for ⟨Δ*E*⟩_d,ref_, *T*_ref_, and *n*, respectively.^65^ The ILT parameters *A*^∞^ and *n*^∞^ used to model barrierless reaction steps were optimized using a Marquardt least-squares fitting algorithm, as described in the Supporting Information. Additionally, some reaction steps along the reaction PES are dissociation pathways (product-forming pathways) that do not possess a barrier. A similar modified Arrhenius expression is used, termed the reverse ILT method in MESMER.

For reaction steps that require a bimolecular collision under pseudo-first-order conditions, one reactant must be defined as deficient (CH) and one as excess (OCS) in MESMER, and the excess reactant concentration must be included in the MESMER input file. For the excess reactant concentration, i.e., the concentration of OCS, a value of 10^9^ cm^–3^ was used for all simulations to examine both the pressure and temperature dependence of *k*(*T*). During typical temperature-dependent kinetic laboratory experiments, the value of the excess concentration is kept at 0.1–1% of the total density. Pressure-dependent reactions are examined in MESMER by increasing the total density while keeping the excess reagent concentration constant. These considerations were taken into account when simulating reaction rate coefficients for the title reaction.

Finally, the overall reaction rate coefficients were determined by dividing the total CH loss rate from the MESMER output by the excess reactant concentration. Product branching fractions as a function of temperature and total density were obtained by retrieving the long-time concentration values for each simulation. The raw data from MESMER simulations can be found in the Supporting Information.

### Classical Capture Theory Calculations

The rate coefficients calculated from statistical rate theory simulations were also compared to those calculated from classical capture theory (CCT). At the collision limit, the rate of a chemical reaction is equal to the rate of collisions between the reacting species, providing an upper limit for the temperature-dependent rate coefficient. The rate coefficient for a bimolecular reaction can be described by eq 5, where *k*(*T*) is the calculated reaction rate coefficient (cm^3^ s^–1^), σ(*T*) is the collision cross section (cm^2^), and ⟨*v*(*T*)⟩ is the average relative molecular velocity (cm s^–1^), with each term dependent on temperature
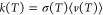
5For hard-sphere collisions, collision theory predicts that *k*(*T*) ∝ *T*^1/2^ since ⟨*v*(*T*)⟩ ∝ *T*^1/2^ and σ(*T*) has no temperature dependence for hard-sphere collisions. However, CCT considers the long-range attractive intermolecular potential between the reacting species. At low temperatures and for a given pressure, the molecules possess a lower average velocity and there are more frequent collisions between the molecules as the total density is higher. The types of attractive intermolecular forces that provide significant contribution to the long-range potential between the reacting species are the dipole–dipole (D–D), dipole–induced-dipole (D–iD), and London dispersion (Disp) intermolecular forces. The CCT rate coefficient (eq 5) can then be transformed into eq 6, where *k*_*B*_ is the Boltzmann constant, *T* is the temperature, Γ(*x*) represents the γ function (Γ(2/3) = 1.353), and μ is the reduced mass of the reacting species
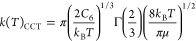
6*C*_6_ in the above equation is a coefficient described by the sum of coefficients relating to the contribution from each of the attractive intermolecular potentials and is represented by eq 7
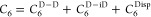
7Breaking this down further, *C*_6_^D–D^ is given by eq 8, where μ_1_ and μ_2_ represent the dipole moment of reacting partners 1 and 2, respectively, and ε_0_ is the vacuum permittivity constant
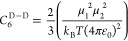
8and *C*_6_^D-iD^ is given by eq 9, where α_1_ and α_2_ represent the volume polarizability of reacting partners 1 and 2, respectively
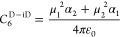
9Finally, *C*_6_^Disp^ is given by eq 10, where *I*_1_ and *I*_2_ represent the ionization energy of reacting partners 1 and 2, respectively
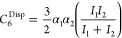
10The reaction rate coefficients predicted by CCT were calculated using the values given in Table 2.

**Table 2:** Parameters Used in the Calculation of *k*(*T*)_CCT_ for the Reaction between CH and OCS

	dipole moment		polarizability	ionization energy	
molecule	Debye	×10^–30^ C m	×10^–30^ m^3^	eV	×10^–18^ J
OCS	0.715^66^	2.38	5.09^67^	11.18^68^	1.79
CH	1.46	4.87^69^	2.40^70^	10.64	1.70^71^

## Results and Discussion

### Approach of CH toward OCS

**Figure 1:**
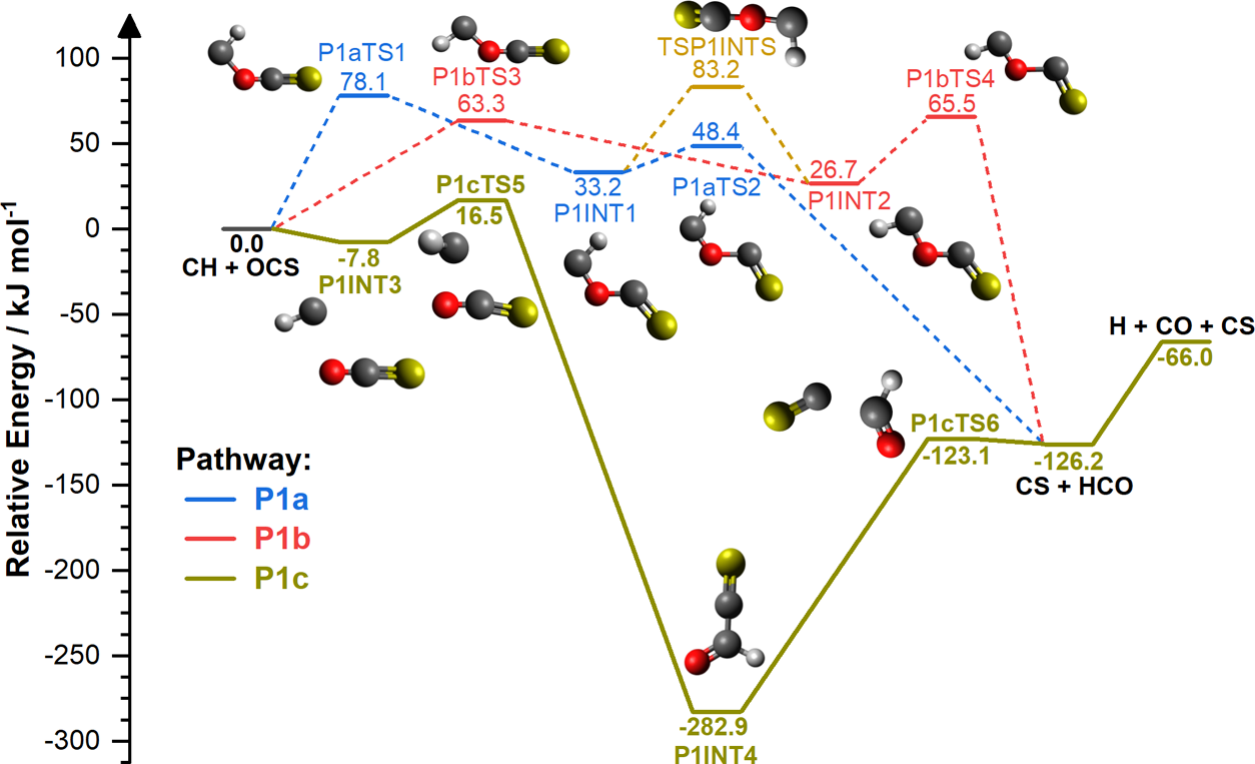
Reaction PES for the approach of CH to the oxygen side of OCS calculated at the ROCCSD(T)/aug-cc-pv(Q+d)Z//M06-2X-D3/aug-cc-pv(Q+d)Z level of theory. Electronic energies (corrected with scaled ZPVE calculated at the M06-2X-D3/aug-cc-pv(Q+d)Z level of theory) are quoted in kJ mol^–1^ relative to reactant species. Bold and dashed lines indicate major and minor reaction pathways, respectively, under the temperature and pressure conditions explored in this work. Blue (P1a), red (P1b), and olive (P1c) pathways show routes to CS + HCO, which can subsequently undergo decomposition to H + CO. Reactants and major reaction products are identified in black bold lettering.

**Figure 2:**
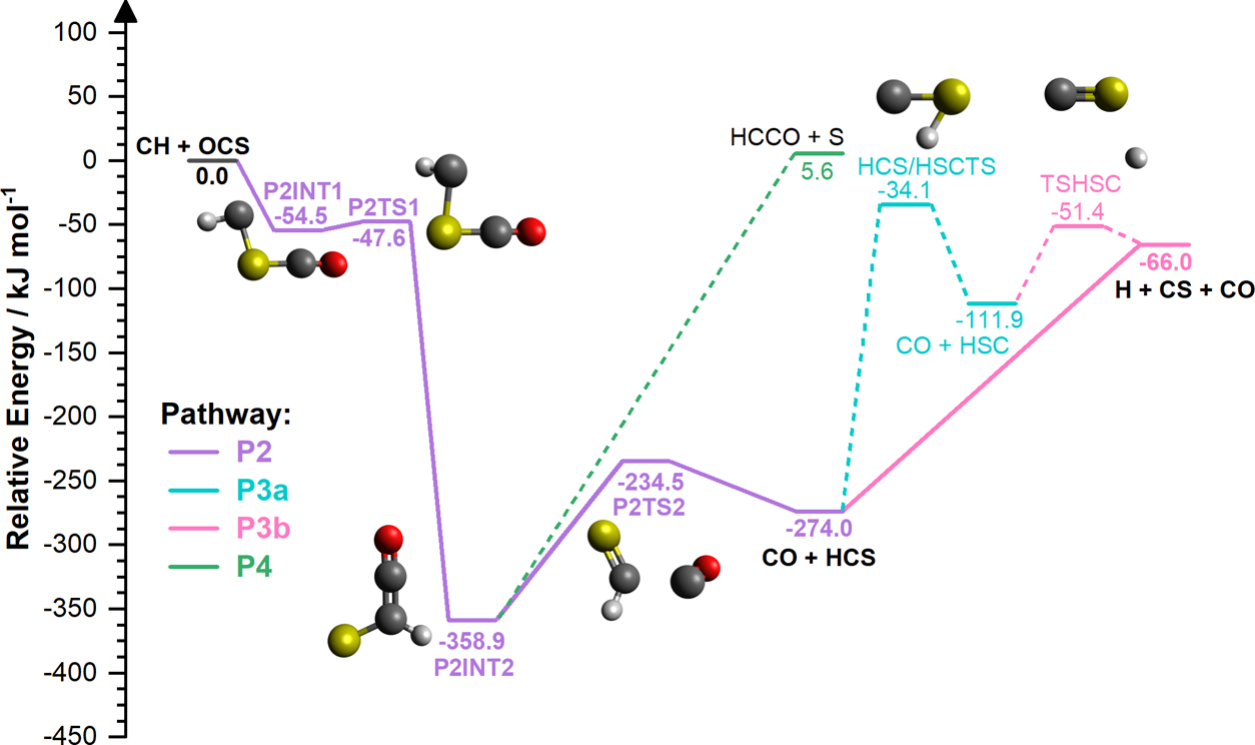
Reaction PES for the approach of CH to the sulfur side of OCS calculated at the ROCCSD(T)/aug-cc-pv(Q+d)Z//M06-2X-D3/aug-cc-pv(Q+d)Z level of theory. Electronic energies (corrected with scaled ZPVE calculated at the M06-2X-D3/aug-cc-pv(Q+d)Z level of theory) are quoted in kJ mol^–1^ relative to reactant species. Bold and dashed lines indicate major and minor reaction pathways, respectively, under the temperature and pressure conditions explored in this work. Note that the energy axis scale is different from Figure 1. The formation of CO + HCS is shown *via* the purple (P2) pathway. Turquoise (P3a) and pink (P3b) pathways show the decomposition of HCS through interconversion or dissociation, respectively. HCCO + S formation is shown by the green (P4) pathway. Reactants and major reaction products are identified in black bold lettering.

Figures 1 and 2 show the calculated reaction PES with the zero-point vibrational energy (ZPVE) corrected electronic energy values quoted in kJ mol^–1^ relative to the CH + OCS entrance channel. Our calculations found three reaction pathways forming CS + HCO, shown in Figure 1, before decomposition of HCO to form H + CO (P1a, blue; P1b, red; P1c, olive), a reaction pathway to CO + HCS, shown in Figure 2, (P2, purple), two reaction pathways to HCS decomposition to form H + CS, shown in Figure 2, (P3a, turquoise; P3b, pink), and a reaction pathway to HCCO + S, shown in Figure 2, (P4, green). These pathways all initially start with CH approaching either the oxygen side, Figure 1, or sulfur side, Figure 2, of OCS leading to different addition complexes in the first instance.

First considering the approach of CH toward the oxygen side of OCS, shown in Figure 1, CS + HCO can be produced through three different identified pathways, two of which are addition–elimination pathways and the third is an insertion–elimination pathway. Two high-energy pathways were found *via* oxygen addition (P1a and P1b) that form an initial cis–trans (hook-shaped P1INT1) or trans–trans (W-shaped P1INT2) intermediate, with barriers of 78.1 kJ mol^–1^ (P1aTS1) and 63.3 kJ mol^–1^ (P1bTS3), respectively. Interconversion can occur between P1INT1 and P1INT2 via TSP1INTS; however, the presence of a significant 83.2 kJ mol^–1^ barrier suggests that this is not competitive. Cleavage of the O–C bond then liberates CS + HCO as P1INT1 or P1INT2 pass through barriers of 48.4 kJ mol^–1^ (P1aTS2) or 65.5 kJ mol^–1^ (P1bTS4), respectively. As these pathways possess a significant initial activation energy barrier, they are unlikely to play a role at low temperatures. However, the formation of CS + HCO is also possible via a lower-energy pathway (P1c), that follows an insertion–elimination reaction mechanism. As CH approaches the oxygen side of OCS, an initial weakly bound van der Waals complex (P1INT3) is formed with an energy of −7.8 kJ mol^–1^ below the entrance channel. CH then inserts into the C–O bond of OCS, passing through a 16.5 kJ mol^–1^ energy barrier, to give a much lower-energy insertion intermediate (P1INT4) that is −282.9 kJ mol^–1^ below the entrance channel, and then dissociation to CS + HCO. Finally, HCO can undergo barrierless decomposition to give H and CO (−66.0 kJ mol^–1^).

Our calculations also found that the CH radical can react with OCS following its approach to the sulfur side of OCS, through a three-step addition–insertion–elimination reaction mechanism. Initially, the CH radical adds to the S atom of OCS forming a complex (P2INT1) that is −54.5 kJ mol^–1^ below CH + OCS. Following addition to OCS, a very low-energy intermediate (−358.9 kJ mol^–1^, P2INT2) forms as CH inserts into the C–S bond of OCS via a submerged energy barrier (−47.6 kJ mol^–1^, P2TS1). Elimination of CO then proceeds through P2TS2 to produce HCS (P2). The reaction products HCCO + S can also form through P2INT2 directly (5.6 kJ mol^–1^, Δ*E* = +364.5 kJ mol^–1^) in a barrierless mechanism (P4). Finally, decomposition of HCS was found to either occur through interconversion to the HSC isomer via HCS/HSCTS (P3a) or through dissociation of HCS (P3b, −66.0. kJ mol^–1^, Δ*E* = +208 kJ mol^–1^). Calculated properties (Cartesian coordinates, electronic energies, rotational constants, and vibrational frequencies) of all optimized structures can be found in Tables S4–S7.

### Overall CH Loss Rate

**Figure 3:**
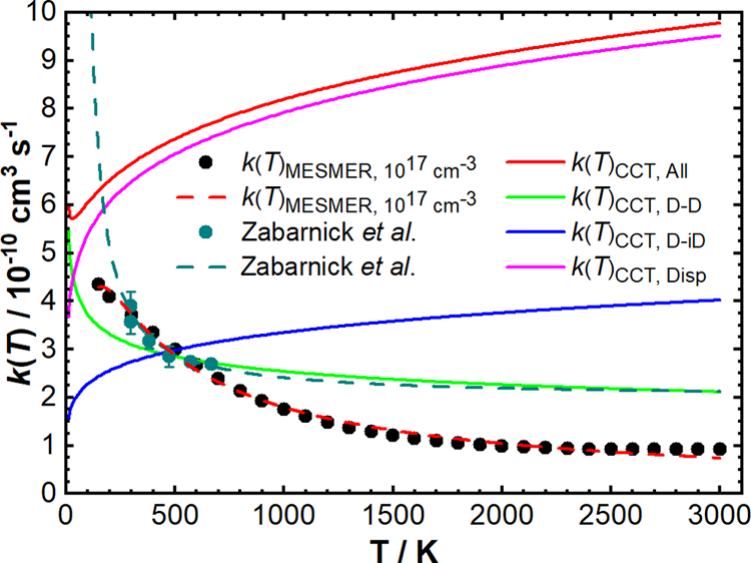
Variation of the experimental and predicted reaction rate coefficients, *k*(*T*), with temperature for the CH (X^2^Π) + OCS reaction. Black circles represent MESMER simulated values of *k*(*T*) at a total density of 10^17^ cm^–3^, with the red dashed line representing a modified Arrhenius fit of *k*(*T*) = (6.09 × 10^–10^) × (T/298)^−0.89^ exp(−1201.4/*RT*) cm^3^ s^–1^. Teal crosses represent experimental data from Zabarnick et al., with the teal dashed line representing the reported Arrhenius fit of *k*(*T*) = (1.99 × 10^–10^) exp(190/*T*) cm^3^ s^–1^.^46^ Solid lines represent values of *k*(*T*) predicted by classical capture theory (CCT) upon inclusion of only dipole–dipole (D–D, green), dipole–induced dipole (D–iD, blue), or London dispersion (Disp, pink) intermolecular forces, respectively. The red solid line represents the CCT value of *k*(*T*) upon inclusion of all intermolecular forces.

The stationary points found from the *ab initio* calculations along the reaction PES for the CH (X^2^Π) + OCS reaction were then utilized in statistical rate theory simulations performed with the MESMER software package. Simulations were performed at 150 K and then in 100 K intervals from 200 to 3000 K over a range of total densities (10^11^–10^24^ cm^–3^). Temperature-dependent reaction rate coefficients for the total loss of CH are overlayed with the experimental values in the temperature range of 297–667 K from the work of Zabarnick et al.^46^ in Figure 3. All MESMER simulated rate coefficients can be found in Tables S8–S10. An Arrhenius function of the form of eq 11 is fit to the experimental rate coefficients in Zabarnick et al.^46^ The rate coefficient, *k*(*T*), is given by the product of the temperature-independent preexponential factor, *A*, and the temperature-dependent exponential term containing the activation energy, *E*_a_, and molar gas constant, *R*.
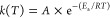
11While the experimental values cover a range of 297–667 K, the MESMER simulation covers a much wider temperature range of 150–3000 K. A modified Arrhenius function, given by eq 12, is a much better descriptor of the simulated rate coefficients over such a wide temperature range, considering the temperature dependence of the *A*-factor
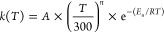
12

The overall reaction rate coefficient simulated by MESMER based on our calculated PES increases with decreasing temperature, starting at 9.21 × 10^–11^ cm^3^ s^–1^ at 3000 K and reaching 4.35 × 10^–10^ cm^3^ s^–1^ at 150 K. Like some other radical-neutral molecule gas-phase reactions,^72^ MESMER simulations predict a negative temperature dependence of *k*(*T*), where the rate coefficient is almost a factor of five greater at 150 K than at 3000 K. This is due to the increase in the association rate coefficient of CH and OCS to give the initial association intermediate P2INT1, which then undergoes rapid insertion into the C–S bond of OCS to give P2INT2. Pressure independence over the range studied here below a total density of 10^22^ cm^–3^ is observed, which suggests that collisional energy transfer through collisions with a third body makes no significant contribution to the overall reaction. Above this total density, however, the increased collision frequency stabilizes the low-energy well, P2INT2, which hinders the formation of reaction products.

### Comparison of Reaction Rate Coefficients with CCT

Experimental and theoretical reaction rate coefficients were then compared to those calculated using CCT, which provides an upper estimate of the overall reaction rate coefficient. Temperature-dependent reaction rate coefficients at a total density of 10^17^ cm^–3^ as predicted by MESMER are shown by the black circles in Figure 3, along with those calculated from CCT (red, green, blue, and pink solid lines) and experimental values determined by Zabarnick et al. in the temperature range of 297–667 K (teal crosses and dashed line).^46^ The CCT values of *k*(*T*) were calculated using eqs 6–10 by considering only dipole–dipole (D–D, green), dipole–induced dipole (D–iD, blue), and London dispersion intermolecular forces (Disp, pink), respectively, before calculating a total *k*(*T*) upon inclusion of these intermolecular forces. All MESMER simulated rate coefficients can be found in Tables S8–S10.

The CCT calculations show a decrease in the value of the upper limit of *k*(*T*) when all intermolecular forces are considered from 9.8 × 10^–10^ cm^3^ s^–1^ at 3000 K to 5.7 × 10^–10^ cm^3^ s^–1^ at 30 K, before increasing again as the temperature continues to decrease. Since the OCS dipole moment is <1 Debye, the long-range attraction of CH and OCS is dominated by London dispersion forces as *C*_6_^Disp^ > *C*_6_^D–D^ down to 30 K. The rate coefficient then increases as the temperature drops below 30 K as the contribution to the rate coefficient from dipole–dipole intermolecular forces becomes greater than those of the London dispersion intermolecular forces. The results from the CCT calculations provide an upper limit on the reaction rate coefficient for a given temperature since CCT calculations exclude short-range interactions and assume that all collisions are “successful,” i.e., go on to form products. Extrapolation of the experimental data to temperatures lower than 170 K results in erroneously large values of the reaction rate coefficient, highlighted by the Arrhenius fit of the experimental data exceeding the CCT results in Figure 3. These nonphysical results point to caution being required for extrapolation of these kinetic data to higher or lower temperatures, and for the need for further experimental data before the behavior at low temperatures is to be understood. Although the MESMER simulations are based on fitted ILT parameters to the experimental data, they do not quite replicate the curvature of the experimental data, and so care must also be taken if attempting to extrapolate the MESMER simulated values as well.

### Product Branching Fractions

**Figure 4:**
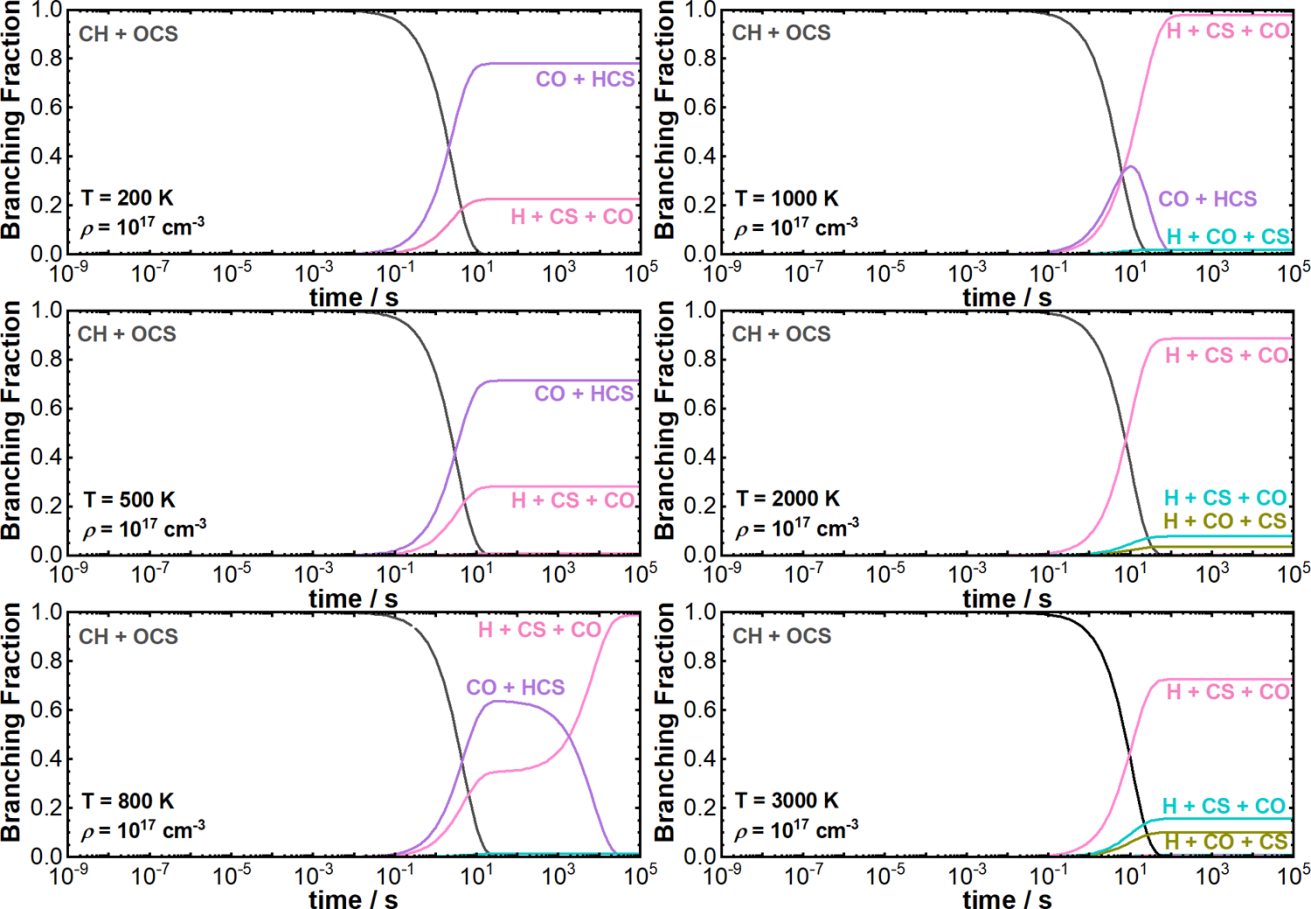
Branching fractions predicted by MESMER as a function of reaction time for temperatures of 200, 500, 800, 1000, 2000, and 3000 K at a total density of 10^17^ cm^–3^. Key: P1c—olive, P2—purple; P3a—turquoise; P3b—pink.

A useful feature of the MESMER software package is that the branching fractions of all minima along the reaction PES can be calculated as the reaction progresses, which can be used to determine the relative contribution each reaction pathway makes to the overall CH loss rate. Product branching fractions from MESMER simulations were determined at 150, 200 K, and then in 100 K intervals up to 3000 K thereafter over a range of total densities (10^11^–10^24^ cm^–3^). The temporal evolution of the molecular species involved in the reaction is shown in Figure 4 for simulations at 200, 500, 800, 1000, 2000, and 3000 K for a total density of 10^17^ cm^–3^.

As is evident in Figure 4, CO + HCS formation is expected to dominate at temperatures below 800 K, accounting for 78.0% of the total CH loss rate at 200 K, but then reducing to 0.0% above 800 K. Our simulations predict that H + CO + CS are the only reaction products above 800 K, which is consistent with the CH + CO_2_ reaction for the H + 2CO product channel. However, the contributing pathways do vary as temperature increases, as shown in Figure 4. The remaining contribution to the total CH loss rate is direct formation of H + CS + CO via pathway P3b, accounting for 21.7% of the CH loss rate at 150 K and increasing to 97.9% at 1000 K. At 1000 K, HCS dissociation (P3b) contributes 97.9% to the formation of H + CO + CS, with a 0.3% contribution from the lower-energy HCO decomposition pathway (P1c), and 1.9% contribution from HSC decomposition (P3a). However, at 3000 K, the contribution from HCS dissociation (P3b) reduces to 72.7% and the contributions from the lower-energy HCO decomposition pathway (P1c), HSC decomposition (P3a), and the higher-energy HCO decomposition pathways (P1a and P1b) increases to 10.1, 15.7, 0.7, and 0.5%, respectively. The remaining 0.2% contribution arises from the formation of HCCO + S (pathway P4).

**Figure 5:**
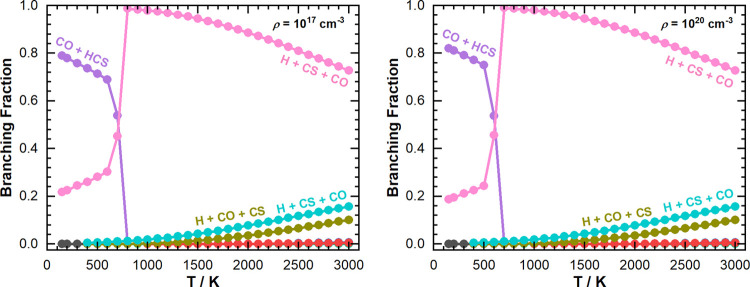
Branching fractions as a function of temperature for total densities of 10^17^ cm^–3^ (left) and 10^20^ cm^–3^ (right), respectively. Key: P1a—blue; P1b—red; P1c—olive, P2—purple; P3a—turquoise; P3b—pink; P4—green.

Another important feature shown in Figure 4 is that the product channels for this reaction change as a function of temperature. For a particular total density, if we plot the branching fraction as a function of temperature (as shown in Figure 5), we can see that there is a temperature at which H + CO + CS become the major reaction products in favor of CO + HCS, which we are calling a “crossover point”. We found that the temperature at which the “crossover point” occurs increases with decreasing total density (as shown in Figure S2). This is further evidence to suggest that at conditions relevant to the ISM, low temperatures, and total densities, CO + HCS are the major reaction products. Additionally, this trend suggests that at higher total densities, there are a greater number of collisions promoting the dissociation of HCS, but dissociation can only take place after HCS has formed from the reaction.

**Figure 6:**
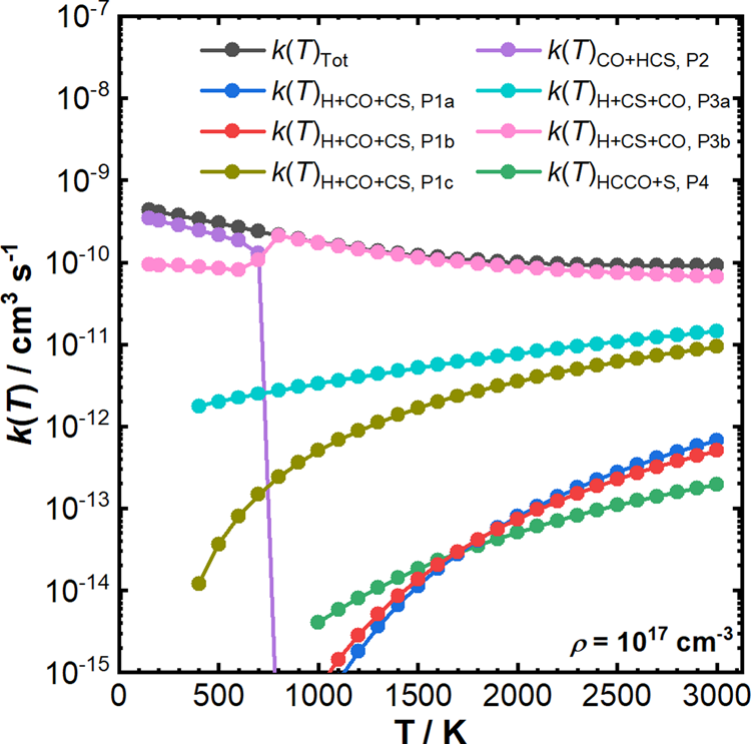
MESMER simulated reaction rate coefficients for the total loss of CH (black) and individual reaction pathways over the temperature range of 150–3000 K and at a total density of 10^17^ cm^–3^. Key: P1a—blue; P1b—red; P1c—olive, P2—purple; P3a—turquoise; P3b—pink; P4—green. See text for further discussion of the trends shown here, particularly for P2 and P3b.

Also, a prominent feature of the simulations at 800 and 1000 K in Figure 4 is the equilibration of HCS that occurs before HCS decomposition. These simulations highlight that the mechanism of formation of H + CO + CS from the title reaction proceeds through HCS formation first. That is, the mechanism of the reaction is that as CH approaches OCS, CH must insert into the C–S bond of OCS to form the insertion intermediate, followed by cleavage of the C–C bond in P2INT2 that releases CO + HCS from which HCS decomposition can then occur. Further analysis of the species’ time profiles then allows for the calculation of reaction pathway-specific rate coefficients. The branching fractions at long reaction times (10^5^ s) were then used to calculate the reaction pathway-specific rate coefficients presented in Figure 6.

Figure 6 shows a general increase in the overall reaction rate coefficient (black) as the temperature decreases, with *k*(*T*) increasing from 9.2 × 10^–11^ cm^3^ s^–1^ at 3000 K to 4.3 × 10^–10^ cm^3^ s^–1^ at 150 K. At 3000 K, CH + OCS reacts ∼2 orders of magnitude slower to produce of H + CO + CS via the higher-energy oxygen addition pathways (P1a and P1b), and almost 3 orders of magnitude slower to produce HCCO + S (P4), compared to the low-energy sulfur addition pathway to dissociation of HCS (P3b). The rate coefficient for those pathways also shows a positive temperature dependence, hence, these pathways are not expected to be competitive over the entire temperature range. Additionally, H + CS + CO formation through the lower-energy oxygen addition pathway (P1c) and through interconversion of HCS to HSC (P3a) is only expected to have a minor contribution to the total loss of CH in the presence of OCS above 900 K. This is because the initial reaction complex (P2INT1) as CH approaches the sulfur side of OCS is more stable than the van der Waals complex (P1INT3) formed as CH approached the oxygen side of OCS, meaning formation of P2INT1 is more favorable at lower temperatures, therefore leading to H + CS + CO formation primarily through formation CO + HCS (P2) before dissociation of HCS (P3b).

The trends shown in Figure 6 and discussed above are also highlighted by the final product branching fractions as a function of temperature and total density as predicted by MESMER (Figures 5 and S2). In addition, examining the branching fractions as a function of temperature and density also showed that the temperature at which decomposition of HCS becomes more important than the formation of HCS decreases with increasing total density. For instance, the temperature at which 50% of the HCS molecules have decomposed to H + CS is ∼710 K for a total density of 10^17^ cm^–3^, in contrast to ∼610 K for a total density of 10^20^ cm^–3^. This trend is likely a result of the rate of HCS decomposition being pressure-dependent.

### Comparison to the CH + CO_2_ System

As sulfur and oxygen are valence isoelectronic, a good system for comparison is that of the CH + CO_2_ reaction, as stated previously.^47^ The first obvious comparison is that a greater number of reaction pathways are possible for the CH + OCS reaction because of the asymmetrical nature of OCS compared to CO_2_. That said, there are a number of comparable features of the reaction mechanism that exist for both systems, particularly for the approach of CH to the oxygen side of OCS. In both cases, HCO formation either occurs *via* insertion of CH into the C–O bond, or addition of CH to the oxygen atom in the first instance. Overall, the structures of P1INT2, P1INT3, and P1INT4 are comparable to IM4, pre-complex, and IM2 of the CH + CO_2_ reaction, respectively, which are presented in Figure 1 of the work by Vichietti et al.,^47^ and similar transition states along the reaction coordinate are also observed for both reactions. However, the mechanism for forming these structures is different between the two systems. In the previous work on CH + CO_2_, both the insertion and addition pathways proceeded via an initial pre-reaction complex. However, for CH + OCS, two high-energy reaction pathways are identified for the addition of CH to the oxygen side of OCS, neither of which relies on the initial formation of an association complex. The third pathway (P1c) is more comparable to the previously reported CH + CO_2_ mechanism in that it proceeds via initial complex formation before the insertion of CH into the C–O bond.

As stated in the Introduction section, replacement of an oxygen atom in CO_2_ by a sulfur atom to give OCS increases the number of possible reaction products, namely, the thioformyl radical (HCS) and its constitutional isomer, HSC. Exploration of HCS isomerization and dissociation was recently prompted by the observation of both the HCS and HSC isomers in the L483 molecular cloud in a 40:1 ratio.^38^ Here, HSC was calculated to be 162.1 kJ mol^–1^ less stable than HCS, which is in agreement with the 164.4 kJ mol^–1^ difference calculated by Puzzarini. Several other groups also calculate this difference to be 159,^51^ 161,^48^ and 166 kJ mol^–1^,^49^ again in excellent agreement with our calculations. Good agreement is also observed for the barrier to HCS isomerization calculated in this study (77.8 kJ mol^–1^ above HSC) and by Puzzarini (83.6 kJ mol^–1^ above HSC). Our barrier is in excellent agreement with the 77 and 76 kJ mol^–1^ barrier calculated by Yamada et al.^51^ and Galland et al.,^48^ respectively. In addition, our calculations also show similar features to those of previous studies for the decomposition of HCS and HSC to H + CS. We calculate that Δ*E*_HCS → H + CS_ = +208 kJ mol^–1^ and Δ*E*_HSC → H + CS_ = +45.9 kJ mol^–1^. This compares well with the 206, 201, and 198 kJ mol^–1^ calculated values for Δ*E*_HCS → H + CS_ and the 42, 42, and 37 kJ mol^–1^ calculated values for Δ*E*_HSC → H + CS_ calculated by Puzzarini, Yamada et al., and Galland et al., respectively. Galland et al. report a small barrier to HCS decomposition, which contrasts with the barrierless breakup observed in calculations performed by Puzzarini and Yamada et al., and now our calculations. In contrast to the HCS and HSC isomers, the HOC isomer of HCO has not been observed in the ISM. The possibility of isomerization of HCO was not included in our study as it has a significant barrier of 107 kJ mol^–1^ reported by Marenich and Boggs^73^ for conversion of HCO to HOC, meaning that under interstellar conditions, this process is unlikely to be relevant. In addition, HOC was also found to be only metastable and 40 kJ mol^–1^ above HCO,^74^ making the formation of CS + HOC from the CH + OCS reaction energetically unfavorable.

The reactivity of CH + OCS is similar to the reactivity of CH + CO_2_, where variational transition state theory was used to determine the equilibrium concentration of stable species during the progress of the reaction. Vichietti et al. also predicted that CO + HCO (−272.0 kJ mol^–1^ below the CH + CO_2_ entrance channel) formation was expected to dominate below 300 K, while H + 2CO (−205.9 kJ mol^–1^ below the CH + CO_2_ entrance channel) became the major reaction products above room temperature.^47^ In contrast, H + CS + CO are the dominant reaction products of the CH + OCS reaction above 800 K, a higher temperature than for the CH + CO_2_ reaction. A review by Loison et al. on the gas-phase reactivity of OCS assumes that the sole reaction products of the title reaction are H + CO + CS based on the highly exothermic CO + HCS and CS + HCO product channels and the weak nature of the H–CO and H–CS bonds.^45^ The aforementioned assumption, coupled with the large negative dependence observed from the experimental work by Zabarnick et al., prompted the KIDA database to recommend a rate coefficient of *k*(*T*) = 4.0 × 10^–10^ cm^3^ s^–1^ for the temperature range of 10–300 K with the reaction products being H + CO + CS. However, our results contradict the suggested reaction pathway listed in KIDA. This contradiction is not so surprising considering CO + HCO are listed as the primary reaction products from 10 to 800 K for the CH + CO_2_ reaction, and therefore one would expect similar reaction products for the CH + OCS reaction. The fact CO + HCS dominates below 700 K can be understood from our *ab initio* calculations, in which no direct route to H + CO + CS formation is predicted. Therefore, to produce H + CS + CO from this reaction, one must allow for the formation of HCS prior to HCS decomposition. This is highlighted by the purple curves at 800 and 1000 K in Figure 4, which shows the equilibration of HCS prior to dissociation. A minor contribution of pathway P3a to form H + CS + CO through isomerization of HCS to HSC was also predicted, with the branching fraction increasing from 0.2% at 1000 K to 15.7% at 3000 K. This reaction pathway has a smaller contribution because of the large barrier to isomerization of HCS, and the fact that HSC is much higher in energy than HCS, which inhibits this reaction channel at lower temperatures. No contribution of HCCO + S is predicted from our simulations, likely due to more exothermic reaction routes being available. However, a full reaction dynamics study alongside experimental product branching ratios would be required to confirm these conclusions.

## Conclusions

In this article, we have presented the first theoretical study of the CH (X^2^Π) + OCS gas-phase reaction using a combination of *ab initio* calculations and statistical rate theory simulations. First, our *ab initio* approach found reaction pathways to five exothermic product channels shown in Figures 1 and 2, that follow a two-step addition–elimination or insertion–elimination reaction mechanism, or a three-step addition–insertion–elimination reaction mechanism as CH approached the O or S atom of OCS, respectively. We have also presented evidence to suggest that the reaction products of H + CO + CS are only formed through dissociation of HCO or HCS and no direct route to the three-body product channel was found. Furthermore, we found good agreement when comparing our theoretical work with previous studies on the CH + CO_2_ reaction and on HCS to HSC isomerization.

Our subsequent rate theory simulations found the reaction rate coefficient has a negative temperature dependence, typical of radical–molecule reactions with a submerged barrier. The overall reaction rate coefficient is controlled by the association of CH and OCS, which suggests that the insertion of CH into the C–S bond, or C–O bond to a lesser extent, is the rate-determining step. In addition, we found that H + CO + CS are the major reaction products above 800 K and 10^17^ cm^–3^ with only the contribution of the reaction pathways forming those products changing with temperature as discussed in the Product Branching Fractions section of this article. We have also presented evidence that below 700 K and 10^17^ cm^–3^, the major reaction products are CO + HCS, with the branching fraction decreasing from 79% at 150 K to 0% at 800 K. Complementary to this is the increase in the branching fraction of H + CO + CS from 22% at 150 K to 100% at 800 K. While we recognize the higher pressure and temperature range under which these MESMER simulations are performed compared to some ISM conditions, our conclusion contradicts the findings of Loison et al. and the recommendations contained in the KIDA database. Loison et al. suggest that H + CO + CS are the sole reaction products in the temperature range of 10–300 K without any evidence from statistical energy distributions during the course of a reaction, or insight from reaction dynamics studies.

In the absence of experimental data over a wide temperature and pressure range, fitted parameters from 297 to 667 K for the ILT and collisional energy transfer parameters are extrapolated over the wider temperature range for our rate coefficient simulations. Experimental measurements of temperature-dependent reaction rate coefficients and product yields (the latter providing a more significant experimental challenge) would help constrain the fitting parameters further and improve MESMER simulations. Furthermore, reaction rate coefficients calculated in this work should be included in astrochemical models in an attempt to better understand the sulfur reservoirs within the ISM and determine the relative importance of this gas-phase reaction in astrophysical environments. Full reaction dynamics studies, further experimental data, and feeding these new data in astrochemical models are all required in order to fully understand this reactive system and determine the contribution this system has on the sulfur depletion problem in the ISM.

## Supporting Information Available

Comparison of calculated and experimental reaction enthalpies; stationary point geometries, rotational constants, vibrational frequencies, and energies; further MESMER simulation details, collisional energy transfer parameters; all predicted rate coefficients, pressure dependence, product branching fractions, and MESMER data fitting (PDF)
