# Supplementary material for: Ab Initio and Statistical Rate Theory Exploration of the CH (X2Π) + OCS Gas-Phase Reaction
Source: J Phys Chem A. 2023 Jul 28;127(31):6509–20. doi: 10.1021/acs.jpca.3c01082 (PMC10424238; doi:10.1021/acs.jpca.3c01082)
Supplement: Supplementary file 1 — jp3c01082_si_001.pdf [file jp3c01082_si_001.pdf]

# Supporting Information:

# An *Ab Initio* and Statistical Rate Theory Exploration of the CH (X $^2\Pi$) + OCS Gas-Phase Reaction

Daniel I. Lucas,[a] Casey J. Kavaliauskas,[b] Mark A. Blitz,[b,c] Dwayne E. Heard,[b] and Julia H. Lehman[a,*]

[a] School of Chemistry, University of Birmingham, Edgbaston, United Kingdom, B15 2TT

[b] School of Chemistry, University of Leeds, Leeds, United Kingdom, LS2 9JT

[c] National Centre for Atmospheric Science, University of Leeds, Leeds, United Kingdom, LS2 9JT

*Corresponding Author: Julia H. Lehman, j.lehman@bham.ac.uk

# Calculated Reaction Enthalpies

Experimental enthalpies of formation at 298 K for all reagent and product species are given in Table S1. These values were then used to calculate the experimental enthalpies of reaction given in Table S2.

**Table S1.** Experimental enthalpies of formation at 298 K ($\Delta \boldsymbol{H}_f^{\mathbf{298\,K}}$).

| Molecule | $\Delta H_f^{298\,K}$ / kJ mol$^{-1}$ | Reference |
|---|---|---|
| CH | 594.1 ± 17.5 | [1] |
| OCS | -138.4 ± 1.1 | [1] |
| CO | -110.5 ± 0.2 | [1] |
| CS | 280.3 ± 25.0 | [1] |
| HCO | 43.5 ± 8.0 | [1] |
| HCS | 305.0 ± 8.4 | [2, 3] |
| HCCO | 176.6 ± 2.9 | [4] |
| S | 277.0 ± 2.9 | [1] |
| H | 218.0 ± 0.0 | [1] |

[1] Chase; [2] Butler & Baer; [3] Butler *et al.*; [4] Osborn;

Table S2 shows the comparison between the calculated enthalpies of reaction, determined from the M06-2X/6-311++G(2d,p) and M06-2X-D3/aug-cc-pV(Q+d)Z levels of theory, and those determined from experimental enthalpies of formation presented in Table S1. All reaction enthalpies were calculated using a Hess cycle approach given by eq 1.

$$\Delta \boldsymbol{H}_{rxn}^{\mathbf{298\,K}} = \sum \Delta H_{products} - \sum \Delta H_{reactants} \tag{1}$$

**Table S2.** Comparison of reaction enthalpies at 298 K ($\Delta \boldsymbol{H}_{rxn}^{\mathbf{298\,K}}$) in kJ mol$^{-1}$ calculated at the M06-2X/6-311++G(2d,p) and M06-2X-D3/aug-cc-pV(Q+d)Z levels of theory in this work with those derived from experimental enthalpies of formation at 298 K ($\Delta \boldsymbol{H}_f^{\mathbf{298\,K}}$). No experimental $\Delta \boldsymbol{H}_f^{\mathbf{298\,K}}$ of HSC is available in the literature.

| Reactions | | | $\Delta H_{rxn}^{298\,K}$ / kJ mol$^{-1}$ | | |
|---|---|---|---|---|---|
| | | | M06-2X[a] | M06-2X-D3[b] | Experiment |
| CH + OCS | → | CS + HCO | -122.3 | -116.9 | -131.9 ± 19.3 |
| | → | CO + HCS | -272.0 | -270.2 | -261.3 ± 19.4 |
| | → | HCCO + S | -12.5 | -4.10 | -2.2 ± 17.8 |
| | → | H + CS + CO | -59.2 | -53.4 | -68.0 ± 30.5 |

[a] Calculations performed at the M06-2X/6-311++G(2d,p) level of theory.

[b] Calculations performed at the M06-2X-D3/aug-cc-pV(Q+d)Z level of theory.

## Optimised Structures – Cartesian Coordinates, Rotational Constants, Vibrational Frequencies, and Electronic Energies:

**Table S3.** Optimised cartesian coordinates at the M06-2X/6-311++G(2d,p) and M06-2X-D3/aug-cc-pV(Q+d)Z levels of theory of all species along the reaction PES for the CH + OCS reaction.

| Molecule | M06-2X/6-311++G(2d,p) | | | | M06-2X-D3/aug-cc-pV(Q+d)Z | | | |
|---|---|---|---|---|---|---|---|---|
| | **Atom** | **x** | **y** | **z** | **Atom** | **x** | **y** | **z** |
| CH | C | 0.000000 | 0.000000 | 0.159592 | C | 0.0000000000 | 0.0000000000 | 0.1586957139 |
| | H | 0.000000 | 0.000000 | -0.957553 | H | 0.0000000000 | 0.0000000000 | -0.9566567139 |
| OCS | O | 0.000000 | 0.000000 | -1.676714 | C | 0.0000000000 | 0.0000000000 | -0.5270523772 |
| | C | 0.000000 | 0.000000 | -0.528389 | O | 0.0000000000 | 0.0000000000 | -1.6742923758 |
| | S | 0.000000 | 0.000000 | 1.036503 | S | 0.0000000000 | 0.0000000000 | 1.0327447529 |
| CS | C | 0.000000 | 0.000000 | -1.109342 | C | 0.0000000000 | 0.0000000000 | -1.1076519311 |
| | S | 0.000000 | 0.000000 | 0.416003 | S | 0.0000000000 | 0.0000000000 | 0.4143129311 |
| HCO | H | -0.868973 | 1.206460 | 0.000000 | C | 0.0592681785 | 0.5794385572 | 0.0000000000 |
| | C | 0.062070 | 0.580972 | 0.000000 | H | -0.8678111661 | 1.2077359563 | 0.0000000000 |
| | O | 0.062070 | -0.586536 | 0.000000 | O | 0.0637099876 | -0.5862785134 | 0.0000000000 |
| CO | C | 0.000000 | 0.000000 | -0.640469 | C | 0.0000000000 | 0.0000000000 | -0.6397518447 |
| | O | 0.000000 | 0.000000 | 0.480352 | O | 0.0000000000 | 0.0000000000 | 0.4796348447 |
| HCS | H | -0.777616 | 1.773782 | 0.000000 | C | 0.0271529257 | 1.0434484196 | 0.0000000000 |
| | C | 0.035346 | 1.048584 | 0.000000 | H | -0.7735277092 | 1.7779735819 | 0.0000000000 |
| | S | 0.035346 | -0.504080 | 0.000000 | S | 0.0394507835 | -0.5031360150 | 0.0000000000 |
| HSC | H | -1.274396 | -0.708978 | 0.000000 | H | -1.2738817149 | -0.7101597341 | 0.0000000000 |
| | S | 0.057927 | -0.417161 | 0.000000 | S | 0.0563659075 | -0.4117101766 | 0.0000000000 |
| | C | 0.057927 | 1.230593 | 0.000000 | C | 0.0589738074 | 1.2263239107 | 0.0000000000 |
| HCCO | C | -1.252157 | -0.126091 | 0.000000 | C | -1.2485665358 | 0.1129376244 | 0.0000000000 |
| | O | 1.188510 | 0.007659 | 0.000000 | O | 1.1897402762 | 0.0024320311 | 0.0000000000 |
| | C | 0.023191 | 0.035827 | 0.000000 | C | 0.0257708484 | 0.0366885690 | 0.0000000000 |
| | H | -2.134286 | 0.480311 | 0.000000 | H | -2.1416865888 | 0.4715235364 | 0.0000000000 |

| | | | | | | | | |
|---|---|---|---|---|---|---|---|---|
| P1aTS1 | C | 2.273887 | 0.396176 | -0.057315 | C | 2.2945084159 | 0.3617527487 | -0.0593015427 |
| | H | 1.769384 | 1.346966 | 0.224961 | H | 1.8363314440 | 1.3346257289 | 0.2190466915 |
| | C | -0.041355 | -0.273529 | 0.17323 | C | 0.0475424463 | -0.2542880256 | 0.1727107188 |
| | O | 1.140353 | -0.518093 | -0.045625 | O | 1.1354090769 | -0.4943818254 | 0.0426791595 |
| | S | -1.517962 | 0.128868 | -0.034715 | S | -1.5250884905 | 0.1234763734 | -0.0348787080 |
| P1INT1 | C | 2.304651 | 0.076241 | 0.000001 | C | 2.3042508342 | 0.0768264761 | 0.0000002597 |
| | H | 2.152064 | 1.178234 | 0.000001 | H | 2.1553252546 | 1.1778576566 | 0.0000007759 |
| | C | -0.014610 | 0.354091 | -0.000002 | C | 0.0180442538 | 0.3485974089 | -0.0000004682 |
| | O | 1.083469 | -0.445432 | -0.000001 | O | 1.0839646288 | -0.4416641309 | 0.0000004852 |
| | S | -1.535004 | -0.012298 | 0.000000 | S | -1.5349264638 | -0.0107814108 | -0.0000010822 |
| P1aTS2 | C | -2.319434 | -0.008839 | 0.000003 | C | -2.3179301570 | -0.0085226719 | 0.0000020476 |
| | H | -2.385184 | 1.096972 | 0.000008 | H | -2.3955321710 | 1.0949600092 | 0.0000059096 |
| | C | 0.110578 | 0.537681 | -0.000005 | C | 0.1190207674 | 0.5385161753 | 0.0000000747 |
| | O | -1.138254 | -0.406943 | -0.000003 | O | -1.1397826210 | -0.4025423024 | -0.0000012447 |
| | S | 1.546522 | -0.063405 | 0.000002 | S | 1.5484521817 | -0.0669452101 | -0.0000017872 |
| P1bTS3 | C | 2.261584 | 0.466151 | 0.023695 | C | 2.2642010077 | 0.4592714214 | 0.0290967604 |
| | H | 3.022674 | -0.284155 | -0.251500 | H | 3.0178883959 | -0.2905999186 | -0.2602926765 |
| | C | -0.067807 | -0.196270 | 0.195362 | C | -0.0693674300 | -0.1918931342 | 0.2027347488 |
| | O | 1.118402 | -0.355293 | -0.053517 | O | 1.1197816930 | -0.3359537435 | -0.0500239119 |
| | S | -1.570784 | 0.094201 | -0.039669 | S | -1.5684346667 | 0.0838093749 | -0.0471439208 |
| P1INT2 | C | 2.232741 | -0.290365 | -0.000006 | C | 2.2340107558 | -0.2892653035 | -0.0000018429 |
| | H | 2.949703 | 0.555870 | -0.000006 | H | 2.9496947061 | 0.5555443772 | -0.0000044615 |
| | C | -0.042226 | -0.398035 | 0.000007 | C | -0.0447534485 | -0.3928455009 | 0.0000015378 |
| | O | 1.077510 | 0.365666 | 0.000004 | O | 1.0782762717 | 0.3620225419 | -0.0000015052 |
| | S | -1.544555 | 0.040575 | -0.000002 | S | -1.5440552850 | 0.0382548853 | 0.0000032719 |
| P1bTS4 | C | -2.286900 | 0.201621 | 0.000006 | C | -2.2874294762 | 0.1974851763 | 0.0000017404 |
| | H | -3.004219 | -0.650449 | 0.000004 | H | -3.0065491189 | -0.6504896006 | 0.0000031892 |
| | C | 0.180649 | 0.616244 | -0.000004 | C | 0.1849994934 | 0.6170872796 | -0.0000011893 |
| | O | -1.160708 | -0.311564 | -0.000005 | O | -1.1614204675 | -0.3076915499 | 0.0000009712 |

| | | | | | | | | |
|---|---|---|---|---|---|---|---|---|
| | S | 1.557961 | -0.110264 | 0.000002 | S | 1.5571825692 | -0.1108033055 | -0.0000017115 |
| TSP1INTS | C | 2.393384 | -0.112114 | -0.109437 | C | 2.3892250000 | -0.1084290000 | -0.1263540000 |
| | H | 2.649214 | -0.257383 | 0.962401 | H | 2.6312310000 | -0.4723700000 | 0.8937330000 |
| | C | -0.085060 | 0.427435 | 0.069369 | C | -0.0878890000 | 0.3977620000 | 0.1475940000 |
| | O | 1.083797 | -0.002883 | -0.097785 | O | 1.0870000000 | 0.0480600000 | -0.0971930000 |
| | S | -1.573095 | -0.100717 | 0.003768 | S | -1.5709530000 | -0.1030070000 | -0.0152270000 |
| P1INT3 | C | -2.510307 | -0.838716 | 0.000002 | C | -2.4979371463 | -0.8273525093 | -0.0000033321 |
| | H | -3.236429 | 0.007041 | -0.000017 | H | -3.2260637781 | 0.0132215106 | -0.0000101190 |
| | C | 0.134747 | 0.382461 | 0.000001 | C | 0.1281976521 | 0.3748920233 | -0.0000004788 |
| | O | -0.912728 | 0.870656 | 0.000001 | O | -0.9232149909 | 0.8525422224 | -0.0000026577 |
| | S | 1.549476 | -0.264672 | -0.000001 | S | 1.5437772632 | -0.2565332469 | 0.0000025876 |
| P1cTS5 | C | 0.085214 | 0.311501 | -0.011187 | C | 0.0777676812 | 0.3127741350 | -0.0104305817 |
| | S | -1.397047 | -0.163599 | 0.013305 | S | -1.3975079025 | -0.1583170023 | 0.0218397436 |
| | O | 1.110829 | 0.897505 | -0.016491 | O | 1.1165059865 | 0.8728875964 | -0.0232690751 |
| | C | 1.762596 | -0.948708 | -0.130419 | C | 1.7827002189 | -0.9302078196 | -0.1449424108 |
| | H | 2.379268 | -0.739222 | 0.768693 | H | 2.3613940158 | -0.7396599095 | 0.7807033240 |
| P1INT4 | C | 0.095853 | 0.179994 | 0.000000 | C | 0.0958984373 | 0.1760931258 | 0.0000000000 |
| | S | 1.602630 | -0.091420 | 0.000000 | S | 1.5986571234 | -0.0838496944 | 0.0000000000 |
| | O | -2.123981 | -0.462885 | 0.000000 | O | -2.1283202294 | -0.4591098432 | 0.0000000000 |
| | C | -1.280554 | 0.430816 | 0.000000 | C | -1.2799959743 | 0.4269016256 | 0.0000000000 |
| | H | -1.542030 | 1.500939 | 0.000000 | H | -1.5343213570 | 1.4974087862 | 0.0000000000 |
| P1cTS6 | C | 1.716475 | 0.273751 | 0.390823 | C | 0.5934965361 | 0.7075285994 | 0.3486471557 |
| | S | 2.522070 | -0.838984 | -0.280873 | S | 1.7318095275 | -0.2134063378 | -0.0776268667 |
| | O | -1.416673 | 0.002887 | 0.040307 | O | -2.3123210431 | -0.5565852754 | 0.1596407155 |
| | C | -0.613647 | 0.806621 | -0.229577 | C | -1.7784387262 | 0.3656694961 | -0.3143058107 |
| | H | -0.622167 | 1.877070 | 0.080219 | H | -2.1006852944 | 1.4281215177 | -0.2412031939 |
| P2INT1 | C | 0.929791 | -0.007392 | -0.000001 | C | 0.9274417736 | 0.0045462317 | -0.0000003266 |
| | O | 2.021236 | 0.318620 | 0.000000 | O | 2.0266472426 | 0.3028412860 | -0.0000002061 |
| | S | -0.600324 | -0.546426 | 0.000000 | S | -0.6091142665 | -0.5155778910 | -0.0000005139 |

| | | | | | | | | |
|---|---|---|---|---|---|---|---|---|
| | C | -1.597613 | 0.971466 | 0.000000 | C | -1.5937894987 | 0.9519046294 | -0.0000004162 |
| | H | -2.557765 | 0.409411 | -0.000001 | H | -2.5558602509 | 0.4019647439 | -0.0000005372 |
| P2TS1 | C | -0.823411 | -0.093702 | 0.008285 | C | -0.8203229913 | -0.0999465408 | 0.0082469305 |
| | O | -1.893792 | 0.288073 | 0.018898 | O | -1.8946292905 | 0.2679535213 | 0.0182632356 |
| | S | 0.689193 | -0.643290 | -0.008799 | S | 0.7025597889 | -0.6199097486 | -0.0077507896 |
| | C | 1.167552 | 1.235675 | -0.102519 | C | 1.1337752943 | 1.2029756226 | -0.1017439000 |
| | H | 2.058408 | 1.136217 | 0.555015 | H | 2.0353871985 | 1.1567461455 | 0.5388445236 |
| P2INT2 | C | -1.107962 | 0.165114 | -0.000001 | C | -1.1066708381 | 0.1654720469 | -0.0000002001 |
| | O | -2.106395 | -0.393599 | 0.000000 | O | -2.1030724233 | -0.3932347025 | -0.0000005381 |
| | S | 1.428225 | -0.269915 | 0.000000 | S | 1.4228044225 | -0.2686154173 | -0.0000001729 |
| | C | 0.087231 | 0.770637 | 0.000000 | C | 0.0885384434 | 0.7704416843 | 0.0000001871 |
| | H | 0.123955 | 1.852917 | 0.000001 | H | 0.1234543956 | 1.8510903886 | 0.0000007120 |
| P2TS2 | C | -1.522363 | 0.336981 | 0.351022 | C | -1.5159590053 | 0.3351334917 | 0.3514305219 |
| | O | -2.143624 | -0.457125 | -0.168213 | O | -2.1415346398 | -0.4526488969 | -0.1695815046 |
| | S | 1.479842 | -0.304916 | 0.034016 | S | 1.4757717124 | -0.3051630199 | 0.0337147045 |
| | C | 0.367055 | 0.775607 | -0.194857 | C | 0.3691906560 | 0.7753723706 | -0.1909156578 |
| | H | 0.403365 | 1.860127 | -0.135545 | H | 0.3968062767 | 1.8579800545 | -0.1382250640 |
| HCS/HSCTS | C | 0.052651 | 1.217899 | 0.000000 | C | 0.0547535964 | 1.2141811567 | 0.0000000000 |
| | S | 0.052651 | -0.471830 | 0.000000 | S | 0.0507034920 | -0.4652698033 | 0.0000000000 |
| | H | -1.158314 | 0.241888 | 0.000000 | H | -1.1584690884 | 0.2390456466 | 0.0000000000 |
| HSCTS | C | 0.085168 | 1.167807 | 0.000000 | C | 0.0926216452 | 1.1707123086 | 0.0000000000 |
| | S | 0.085168 | -0.366382 | 0.000000 | S | 0.0885700981 | -0.3591355974 | 0.0000000000 |
| | H | -1.873707 | -1.144733 | 0.000000 | H | -1.8845627433 | -1.1548847111 | 0.0000000000 |

**Table S4.** Rotational constants ($cm^{-1}$) and unscaled vibrational frequencies ($cm^{-1}$) calculated at the M06-2X/6-311++G(2d,p) and M06-2X-D3/aug-cc-pV(Q+d)Z levels of theory all species in the reaction of CH + OCS.

| **Molecule** | **M06-2X/6-311++G(2d,p)** | | **M06-2X-D3/aug-cc-pV(Q+d)Z** | |
|---|---|---|---|---|
| | **Rotational Constants / $cm^{-1}$** | **Unscaled Vibrational Frequencies / $cm^{-1}$** | **Rotational Constants / $cm^{-1}$** | **Unscaled Vibrational Frequencies / $cm^{-1}$** |
| CH | 14.518 | 2877.77 | 14.565 | 2883.18 |
| OCS | 0.204 | 535.23, 535.23, 879.66, 2153.93 | 0.205 | 541.45, 541.45, 890.32, 2157.93 |
| CS | 0.830 | 1346.46 | 0.834 | 1357.61 |
| HCO | 23.737, 1.517, 1.426 | 1104.53, 1995.53, 2729.77 | 24.104, 1.519, 1.429 | 1105.81, 1998.12, 2721.65 |
| CO | 1.956 | 2278.87 | 1.961 | 2278.53 |
| HCS | 30.150, 0.687, 0.672 | 835.58, 1234.93, 3137.47 | 31.628, 0.691, 0.676 | 817.98, 1248.93, 3141.58 |
| HSC | 9.870, 0.694, 0.648 | 818.00, 933.63, 2467.49 | 9.915, 0.702, 0.655 | 810.13, 948.69, 2473.07 |
| HCCO | 41.064, 0.367, 0.364 | 395.65, 517.11, 578.43, 1287.92, 2120.87, 3375.63 | 44.687, 0.367, 0.364 | 393.56, 519.33, 580.10, 1297.75, 2128.78, 3389.11 |
| P1aTS1 | 1.703, 0.105, 0.100 | -771.41, 119.35, 362.82, 471.15, 583.67, 770.38, 1386.38, 1656.59, 2910.28 | 1.874, 0.104, 0.099 | -706.22, 103.16, 340.47, 479.78, 596.10, 764.46, 1377.03, 1664.29, 2898.06 |
| P1INT1 | 2.765, 0.104, 0.100 | 95.39, 289.05, 493.50, 657.00, 935.03, 1193.60, 1322.55, 1458.18, 2908.42 | 2.811, 0.104, 0.100 | 91.69, 286.48, 493.58, 666.16, 931.95, 1203.22, 1336.94, 1446.99, 2898.58 |
| P1aTS2 | 2.276, 0.101, 0.096 | -932.86, 70.86, 286.75, 475.52, 719.41, 1180.93, 1255.64, 1439.32, 2934.96 | 2.286, 0.100, 0.096 | -955.31, 72.68, 284.71, 469.84, 722.27, 1178.43, 1262.03, 1442.53, 2926.53 |
| P1bTS3 | 2.776, 0.099, 0.096 | -602.5, 146.64, 387.21, 511.21, 702.74, 735.05, 1399.81, 1643.14, 3009.68 | 2.932, 0.099, 0.097 | -557.12, 151.86, 383.53, 529.97, 734.97, 747.78, 1394.38, 1639.47, 3004.79 |
| P1INT2 | 3.123, 0.103, 0.100 | 93.18, 294.90, 492.89, 642.53, 930.94, 1238.13, 1329.93, 1453.39, 2956.48 | 3.185, 0.103, 0.100 | 85.74, 292.20, 492.99, 632.46, 927.37, 1245.03, 1343.68, 1439.62, 2951.24 |
| P1bTS4 | 2.282, 0.098, 0.094 | -1066.82, 58.43, 269.51, 480.12, 669.69, 1156.98, 1220.76, 1419.62, 2886.38 | 2.294, 0.098, 0.094 | -1092.39, 53.24, 267.81, 475.35, 657.44, 1135.38, 1230.87, 1423.33, 2878.27 |
| TSP1INTS | 4.245, 0.096, 0.095 | -292.36, 146.84, 314.96, 394.02, 747.81, 1136.86, 1350.93, 1577.00, 2915.62 | 4.209, 0.097, 0.096 | -266.15, 146.83, 311.62, 433.71, 738.45, 1136.92, 1343.71, 1594.88, 2907.42 |
| P1INT3 | 0.686, 0.096, 0.084 | 54.56, 56.40, 199.11, 524.89, 527.49, 640.62, 894.40, 2114.71, 2896.21 | 0.713, 0.096, 0.085 | 60.26, 125.48, 193.14, 529.91, 534.23, 686.09, 904.73, 2118.39, 2880.16 |

| | | | | |
|---|---|---|---|---|
| P1cTS5 | 0.624, 0.134, 0.111 | -439.00, 178.53, 196.99, 439.85, 519.41, 850.88, 985.08, 1954.58, 2961.94 | 0.650, 0.133, 0.111 | 413.91, 187.22, 337.30, 453.79, 526.81, 863.81, 1025.60, 1975.57, 2949.62 |
| P1INT4 | 1.978, 0.095, 0.091 | 167.97, 180.49, 587.02, 833.94, 903.00, 1366.53, 1574.97, 1617.97, 3006.11 | 2.018, 0.096, 0.091 | 170.34, 196.48, 590.89, 837.99, 906.50, 1368.66, 1584.31, 1635.50, 2993.40 |
| P1cTS6 | 0.860, 0.074, 0.070 | -225.73, 49.69, 130.43, 166.19, 378.13, 1099.99, 1313.85, 1963.50, 2818.51 | 0.872, 0.073, 0.069 | -206.04, 42.59, 132.56, 166.57, 386.07, 1101.34, 1325.80, 1965.36, 2813.45 |
| P2INT1 | 0.744, 0.135, 0.115 | 129.42, 262.94, 369.74, 369.79, 553.77, 729.14, 1120.55, 2160.05, 2938.15 | 0.802, 0.135, 0.115 | 143.00, 318.19, 359.96, 419.52, 564.32, 729.85, 1107.37, 2154.42, 2960.83 |
| P2TS1 | 0.487, 0.166, 0.124 | -189.78, 252.06, 292.95, 417.39, 489.49, 782.79, 1076.08, 2192.38, 2935.35 | 0.517, 0.166, 0.127 | -229.97, 271.59, 302.29, 409.83, 493.41, 784.11, 1077.68, 2189.66, 2957.90 |
| P2INT2 | 1.075, 0.112, 0.101 | 165.07, 497.81, 602.91, 641.36, 885.88, 1118.07, 1273.20, 2244.97, 3198.29 | 1.077, 0.112, 0.102 | 164.85, 501.55, 605.85, 647.13, 893.69, 1121.26, 1275.79, 2250.19, 3190.77 |
| P2TS2 | 0.811, 0.096, 0.088 | -389.70, 86.86, 239.18, 272.35, 595.90, 982.66, 1168.28, 2121.78, 3165.79 | 0.815, 0.096, 0.088 | -399.80, 88.58, 239.64, 276.82, 607.21, 977.43, 1179.00, 2119.26, 3166.08 |
| HCS/HSCTS | 11.691, 0.674, 0.638 | -1690.02, 890.90, 2298.67 | 11.691, 0.683, 0.645 | -1675.23, 906.36, 2344.067 |
| TSHSC | 4.825, 0.758, 0.655 | -628.14, 280.68, 1296.00 | 4.782, 0.759, 0.655 | -603.18, 272.75, 1311.85 |

**Table S5.** Electronic energies calculated at three levels of theory (energy ZPVE scaled and corrected, Hartree) of all species along the reaction PES.

| **Molecule** | **Electronic Energy (ZPVE Scaled and Corrected) / Hartree** | | |
|---|---|---|---|
| | **M06-2X/6-311++G(2d,p)** | **M06-2X-D3/aug-cc-pV(Q+d)Z** | **ROCCSD(T)/aug-cc-pV(Q+d)Z //M06-2X-D3/aug-cc-pV(Q+d)Z** |
| CH | -38.4643 | -38.4688 | -38.4132 |
| OCS | -511.5175 | -511.5440 | -510.9672 |
| CS | -436.2002 | -436.2146 | -435.7205 |
| HCO | -113.8282 | -113.8428 | -113.7079 |
| CO | -113.3077 | -113.3214 | -113.1850 |
| HCS | -436.7779 | -436.7944 | -113.1850 |
| HSC | -436.7163 | -436.7326 | -113.1850 |

| | | | |
|---|---|---|---|
| S | -398.0960 | -398.1036 | -397.6686 |
| H | -0.4982 | -0.4988 | -0.4999 |
| HCCO | -151.8904 | -151.9106 | -151.7096 |
| P1aTS1 | -549.9585 | -549.9921 | -549.3506 |
| P1INT1 | -549.9725 | -550.0040 | -549.3677 |
| P1aTS2 | -549.9667 | -549.9974 | -549.3619 |
| P1bTS3 | -549.9655 | -549.9988 | -549.3562 |
| P1INT2 | -549.9754 | -550.0065 | -549.3702 |
| P1bTS4 | -549.9612 | -549.9914 | -549.3554 |
| TSP1INTS | -549.9578 | -549.9914 | -549.3487 |
| P1INT3 | -549.9863 | -550.0171 | -549.3833 |
| P1cTS5 | -549.9774 | -550.0090 | -549.3740 |
| P1INT4 | -550.0949 | -550.1263 | -549.4881 |
| P1cTS6 | -550.0292 | -550.0583 | -549.4272 |
| P2INT1 | -550.0028 | -550.0373 | -549.4011 |
| P2TS1 | -550.0013 | -550.0350 | -549.3985 |
| P2INT2 | -550.1272 | -550.1583 | -549.5170 |
| P2TS2 | -550.0745 | -550.1046 | -549.4697 |
| HCS/HSCTS | -436.6851 | -436.7021 | -436.2084 |
| TSHSC | -436.6916 | -436.7072 | -436.2149 |

**Table S6.** Relative energies (kJ $mol^{-1}$) of all stationary points along the reaction PES calculated at three levels of theory.

| **Molecule** | **Relative Energy (ZPVE Scaled and Corrected) / kJ $mol^{-1}$** | | |
|---|---|---|---|
| | **M06-2X/6-311++G(2d,p)** | **M06-2X-D3/aug-cc-pV(Q+d)Z** | **ROCCSD(T)/aug-cc-pV(Q+d)Z//M06-2X-D3/aug-cc-pV(Q+d)Z** |
| CH + OCS | 0.0000 | 0.0000 | 0.0000 |
| P1aTS1 | 61.2356 | 54.4288 | 78.0549 |

| | | | |
|---|---|---|---|
| P1INT1 | 24.4192 | 23.2058 | 33.1790 |
| P1aTS2 | 39.6211 | 40.5487 | 48.4171 |
| P1bTS3 | 42.9739 | 36.8690 | 63.2655 |
| P1INT2 | 16.8864 | 16.5279 | 26.6589 |
| P1bTS4 | 54.0873 | 56.1865 | 65.5370 |
| TSP1INTS | 63.1033 | 56.0779 | 83.1623 |
| P1INT3 | -11.6864 | -11.3394 | -7.7585 |
| P1cTS5 | 11.6191 | 10.0312 | 16.5383 |
| P1INT4 | -296.8643 | -297.9615 | -282.9363 |
| P1cTS6 | -124.3905 | -119.3910 | -123.1027 |
| P2INT1 | -55.0550 | -64.3962 | -54.4860 |
| P2TS1 | -51.0211 | -58.3594 | -47.5954 |
| P2INT2 | -381.6832 | -381.8919 | -358.8844 |
| P2TS2 | -243.3031 | -240.8858 | -234.4925 |
| HCS/HSCTS | -28.7740 | -28.1041 | -34.1409 |
| TSHSC | -45.7692 | -41.5144 | -51.3763 |
| CS + HCO | -122.4411 | -117.1230 | -126.1917 |
| CO + HCS | -272.3383 | -270.5318 | -274.0077 |
| CO + HSC | -110.5627 | -108.3645 | -111.9005 |
| HCCO + S | -12.1522 | -3.7560 | 5.5646 |
| H + CS + CO | -63.6969 | -57.8910 | -65.9592 |

# MESMER Simulations

Specific details of how the calculated reaction rate coefficients were obtained are given in the MESMER methodology section of the main article. In brief, MESMER requires a simple exponential down collisional energy transfer model, and typical collisional energy transfer parameters that are used for simulations are given in Table S7 for He, Ar and $N_2$ buffer gasses. These parameters could, in principle, be optimised through data fitting procedures, but due to the lack of experimental data over a wide temperature range and the pressure independent nature of the reaction, only standard reference values were used for simulations in this work. Simulations were then performed using the input conditions described Table S7 and in the methodology section of the main article to generate the reaction rate coefficients listed in Tables S8-S10. Here, the overall reaction rate coefficients are reported for the entire temperature and total density ranges of the simulations.

**Table S7.** Collisional energy transfer parameters used in MESMER simulations.

| Bath Gas | $\langle \Delta E \rangle_{d,ref}$ / cm$^{-1}$ | $T^{\infty}$ / K | $n^{\infty}$ |
|---|---|---|---|
| He | 100 | 298 | 1.00 |
| Ar | 200 | 298 | 0.50 |
| $N_2$ | 250 | 298 | 0.25 |

**Table S8.** Total CH loss rate coefficients for the reaction of CH + OCS predicted by MESMER simulations over the temperature and total density ranges of 150 – 3000 K and $10^{10}$ – $10^{14}$ cm$^{-3}$, respectively.

| | $k(T)$ / $10^{-10}$ cm$^3$ s$^{-1}$ | | | |
|---|---|---|---|---|
| T / K | $10^{11}$ / cm$^{-3}$ | $10^{12}$ / cm$^{-3}$ | $10^{13}$ / cm$^{-3}$ | $10^{14}$ / cm$^{-3}$ |
| 150 | 4.34795 | 4.34795 | 4.34795 | 4.34795 |
| 200 | 4.09473 | 4.09473 | 4.09473 | 4.09473 |
| 300 | 3.71160 | 3.71160 | 3.71160 | 3.71160 |
| 400 | 3.34527 | 3.34527 | 3.34527 | 3.34527 |
| 500 | 2.99277 | 2.99277 | 2.99277 | 2.99277 |
| 600 | 2.67006 | 2.67006 | 2.67006 | 2.67006 |
| 700 | 2.38510 | 2.38510 | 2.38510 | 2.38510 |

| | | | | |
|---|---|---|---|---|
| 800 | 2.13888 | 2.13888 | 2.13888 | 2.13888 |
| 900 | 1.92899 | 1.92899 | 1.92899 | 1.92899 |
| 1000 | 1.75150 | 1.75150 | 1.75150 | 1.75150 |
| 1000 | 1.75566 | 1.75566 | 1.75566 | 1.75566 |
| 1100 | 1.60498 | 1.60498 | 1.60498 | 1.60498 |
| 1200 | 1.47871 | 1.47871 | 1.47871 | 1.47871 |
| 1300 | 1.37301 | 1.37301 | 1.37301 | 1.37301 |
| 1400 | 1.28460 | 1.28460 | 1.28460 | 1.28460 |
| 1500 | 1.21073 | 1.21073 | 1.21073 | 1.21073 |
| 1600 | 1.14910 | 1.14910 | 1.14910 | 1.14910 |
| 1700 | 1.09780 | 1.09780 | 1.09780 | 1.09780 |
| 1800 | 1.05531 | 1.05531 | 1.05531 | 1.05531 |
| 1900 | 1.02032 | 1.02032 | 1.02032 | 1.02032 |
| 2000 | 0.99176 | 0.99176 | 0.99176 | 0.99176 |
| 2100 | 0.96872 | 0.96872 | 0.96872 | 0.96872 |
| 2200 | 0.95049 | 0.95049 | 0.95049 | 0.95049 |
| 2300 | 0.93644 | 0.93644 | 0.93644 | 0.93644 |
| 2400 | 0.92607 | 0.92607 | 0.92607 | 0.92607 |
| 2500 | 0.91894 | 0.91894 | 0.91894 | 0.91894 |
| 2600 | 0.91469 | 0.91469 | 0.91469 | 0.91469 |
| 2700 | 0.91302 | 0.91302 | 0.91302 | 0.91302 |
| 2800 | 0.91366 | 0.91366 | 0.91366 | 0.91366 |
| 2900 | 0.91639 | 0.91639 | 0.91639 | 0.91639 |
| 3000 | 0.92101 | 0.92101 | 0.92101 | 0.92101 |

**Table S9.** Total CH loss rate coefficients for the reaction of CH + OCS predicted by MESMER simulations over the temperature and total density ranges of 150 – 3000 K and $10^{15}$ – $10^{19}$ $cm^{-3}$, respectively.

| | $\boldsymbol{k(T)}$ / $\mathbf{10^{-10}\ cm^3\ s^{-1}}$ | | | | |
|---|---|---|---|---|---|
| **T / K** | **$10^{15}$ / $cm^{-3}$** | **$10^{16}$ / $cm^{-3}$** | **$10^{17}$ / $cm^{-3}$** | **$10^{18}$ / $cm^{-3}$** | **$10^{19}$ / $cm^{-3}$** |
| 150 | 4.34795 | 4.34795 | 4.34795 | 4.34795 | 4.34797 |
| 200 | 4.09473 | 4.09473 | 4.09473 | 4.09473 | 4.09476 |
| 300 | 3.71160 | 3.71160 | 3.71160 | 3.71160 | 3.71163 |
| 400 | 3.34527 | 3.34527 | 3.34527 | 3.34528 | 3.34535 |
| 500 | 2.99277 | 2.99277 | 2.99277 | 2.99277 | 2.99283 |
| 600 | 2.67006 | 2.67006 | 2.67006 | 2.67006 | 2.67011 |
| 700 | 2.38510 | 2.38510 | 2.38510 | 2.38511 | 2.38515 |
| 800 | 2.13888 | 2.13888 | 2.13888 | 2.13889 | 2.13892 |
| 900 | 1.92899 | 1.92899 | 1.92900 | 1.92900 | 1.92903 |
| 1000 | 1.75150 | 1.75150 | 1.75150 | 1.75151 | 1.75153 |
| 1000 | 1.75566 | 1.75566 | 1.75566 | 1.75566 | 1.75569 |
| 1100 | 1.60498 | 1.60498 | 1.60498 | 1.60498 | 1.60501 |
| 1200 | 1.47871 | 1.47871 | 1.47871 | 1.47871 | 1.47874 |
| 1300 | 1.37301 | 1.37301 | 1.37301 | 1.37301 | 1.37304 |
| 1400 | 1.28460 | 1.28460 | 1.28460 | 1.28460 | 1.28463 |
| 1500 | 1.21073 | 1.21073 | 1.21073 | 1.21073 | 1.21076 |
| 1600 | 1.14910 | 1.14910 | 1.14910 | 1.14910 | 1.14913 |
| 1700 | 1.09780 | 1.09780 | 1.09780 | 1.09780 | 1.09783 |
| 1800 | 1.05531 | 1.05531 | 1.05531 | 1.05531 | 1.05535 |
| 1900 | 1.02032 | 1.02032 | 1.02033 | 1.02033 | 1.02037 |
| 2000 | 0.99176 | 0.99176 | 0.99176 | 0.99176 | 0.99181 |
| 2100 | 0.96872 | 0.96872 | 0.96872 | 0.96873 | 0.96878 |
| 2200 | 0.95049 | 0.95049 | 0.95049 | 0.95049 | 0.95055 |
| 2300 | 0.93644 | 0.93644 | 0.93644 | 0.93645 | 0.93651 |

| | | | | | |
|---|---|---|---|---|---|
| 2400 | 0.92607 | 0.92607 | 0.92607 | 0.92608 | 0.92615 |
| 2500 | 0.91894 | 0.91894 | 0.91894 | 0.91895 | 0.91903 |
| 2600 | 0.91469 | 0.91469 | 0.91470 | 0.91470 | 0.91479 |
| 2700 | 0.91302 | 0.91302 | 0.91302 | 0.91303 | 0.91312 |
| 2800 | 0.91366 | 0.91366 | 0.91366 | 0.91367 | 0.91377 |
| 2900 | 0.91639 | 0.91639 | 0.91639 | 0.91640 | 0.91651 |
| 3000 | 0.92101 | 0.92101 | 0.92101 | 0.92103 | 0.92115 |

**Table S10.** Total CH loss rate coefficients for the reaction of CH + OCS predicted by MESMER simulations over the temperature and total density ranges of 150 – 3000 K and $10^{20}$ – $10^{24}$ cm$^{-3}$, respectively.

| | ***k*(*T*) / $10^{-10}$ cm$^3$ s$^{-1}$** | | | | |
|---|---|---|---|---|---|
| **T / K** | **$10^{20}$ / cm$^{-3}$** | **$10^{21}$ / cm$^{-3}$** | **$10^{22}$ / cm$^{-3}$** | **$10^{23}$ / cm$^{-3}$** | **$10^{24}$ / cm$^{-3}$** |
| 150 | 4.34813 | 4.34978 | 4.36372 | 4.41602 | 4.45164 |
| 200 | 4.09498 | 4.09720 | 4.11657 | 4.20097 | 4.27344 |
| 300 | 3.71194 | 3.71502 | 3.74300 | 3.89459 | 4.09040 |
| 400 | 3.34608 | 3.35331 | 3.42329 | 4.00251 | 7.17339 |
| 500 | 2.99339 | 2.99894 | 3.05258 | 3.49075 | 6.24850 |
| 600 | 2.67057 | 2.67517 | 2.71965 | 3.08931 | 5.31523 |
| 700 | 2.38554 | 2.38944 | 2.42731 | 2.75054 | 4.72289 |
| 800 | 2.13926 | 2.14264 | 2.17547 | 2.46358 | 4.29777 |
| 900 | 1.92933 | 1.93232 | 1.96132 | 2.22161 | 3.98325 |
| 1000 | 1.75181 | 1.75453 | 1.78067 | 2.01842 | 3.75215 |
| 1000 | 1.75596 | 1.75861 | 1.78414 | 2.01648 | 3.72187 |
| 1100 | 1.60527 | 1.60776 | 1.63135 | 1.84619 | 3.55774 |
| 1200 | 1.47900 | 1.48141 | 1.50375 | 1.70507 | 3.44402 |
| 1300 | 1.37330 | 1.37572 | 1.39744 | 1.58911 | 3.37051 |
| 1400 | 1.28491 | 1.28739 | 1.30905 | 1.49489 | 3.17118 |
| 1500 | 1.21106 | 1.21367 | 1.23576 | 1.41941 | 3.01443 |

| | | | | | |
|---|---|---|---|---|---|
| 1600 | 1.14944 | 1.15225 | 1.17521 | 1.36008 | 2.90149 |
| 1700 | 1.09816 | 1.10109 | 1.12543 | 1.31470 | 2.82868 |
| 1800 | 1.05569 | 1.05876 | 1.08488 | 1.28148 | 2.79221 |
| 1900 | 1.02073 | 1.02398 | 1.05194 | 1.25887 | 2.78841 |
| 2000 | 0.99220 | 0.99566 | 1.02500 | 1.24559 | 2.81418 |
| 2100 | 0.96922 | 0.97294 | 1.00400 | 1.24063 | 2.86690 |
| 2200 | 0.95104 | 0.95519 | 0.98818 | 1.24313 | 2.94448 |
| 2300 | 0.93706 | 0.94172 | 0.97693 | 1.25213 | 3.04524 |
| 2400 | 0.92676 | 0.93205 | 0.97090 | 1.26128 | 3.16782 |
| 2500 | 0.91971 | 0.92569 | 0.96961 | 1.27657 | 3.29479 |
| 2600 | 0.91556 | 0.92227 | 0.97166 | 1.29759 | 3.29747 |
| 2700 | 0.91398 | 0.92147 | 0.97673 | 1.32400 | 3.26336 |
| 2800 | 0.91473 | 0.92305 | 0.98459 | 1.35884 | 3.23512 |
| 2900 | 0.91757 | 0.92678 | 0.99500 | 1.40180 | 3.21460 |
| 3000 | 0.92231 | 0.93247 | 1.00777 | 1.44910 | 3.19978 |

The values in Table S8-S10 above are presented in Figure S1 below, highlighting the pressure independent nature of the reaction (Figure S1) over the total densities of $10^{11} – 10^{21}$ cm$^{-3}$. Figure S1 depicts a clear increase in the overall reaction rate coefficient as the temperature decreases for the total density range of $10^{11} – 10^{21}$ cm$^{-3}$. This trend is also observed for many other radical + neutral molecule reactions. In addition, the pressure independent nature of the title reaction is clearly shown here up to a total density of $10^{21}$ cm$^{-3}$. Whilst there is very slight deviation in the reaction rate coefficients for the simulations above 1000 K at total densities of $10^{20}$ and $10^{21}$ cm$^{-3}$, the increase in the value of $k(T)$ is smaller than the numerical accuracy at which the simulations were performed. For pressure dependent reactions, reaction rate coefficients can be estimated at the high-pressure and low-pressure limits by increasing or reducing the total density of the simulation until no change in the reaction rate coefficient is observed. However, this reaction is observed to be pressure independent as reducing the total density from $10^{21}$ cm$^{-3}$ to $10^{11}$ cm$^{-3}$ resulted in only a very small decrease in the reaction rate coefficient, from $1.76 \times 10^{-10}$ cm$^3$ s$^{-1}$ to $1.75 \times 10^{-10}$ cm$^3$ s$^{-1}$, respectively. Increasing the total density of the simulation resulted in stabilisation of the low energy intermediate (P2INT2 of Figure 2 in the main article).

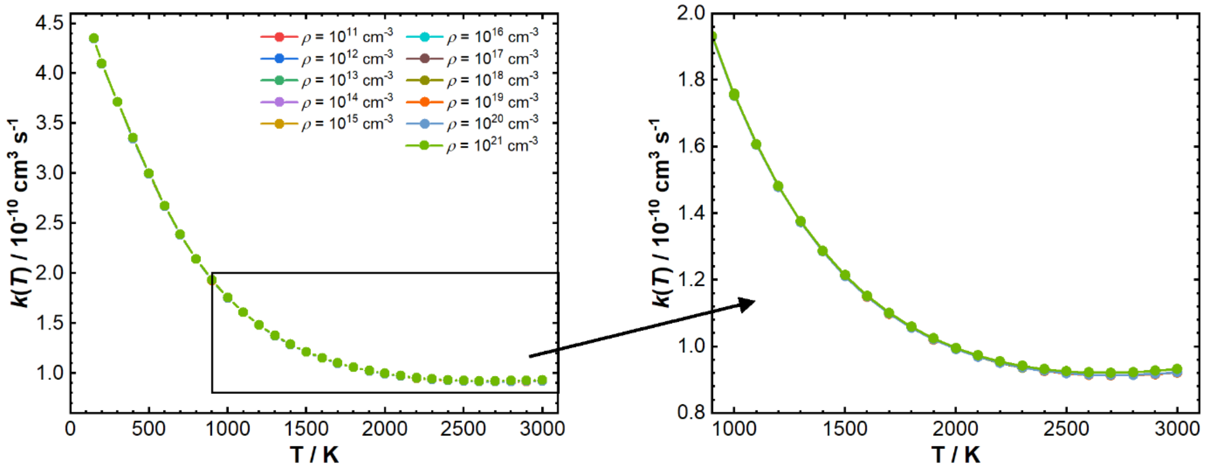


**Figure S1.** Simulated overall reaction rate coefficients in the temperature range of 150 – 3000 K for total densities of $10^{11}$ – $10^{21}$ $cm^{-3}$, highlighting the pressure independence of the reaction. Note that the green trace is the only viewable color because all traces are overlapped.

In addition to the simulation of temperature and pressure dependent reaction rate coefficients, we were also able to determine product branching fractions as a function of temperature and total density, as seen in the main text Figure 5. Interestingly, CO + HCS formation is expected to dominate at temperatures below 700 K, accounting for 79% of the total CH loss rate at 150 K, but then reducing to 0% above 800 K. Our simulations predict that H + CO + CS are the only reaction products above 700 K, which is similar to the dominance of the H + 2CO product channel in the CH + $CO_2$ reaction. However, the contributing pathways do vary as temperature increases, as shown in Figures 5 and 6 in the main text. The remaining contribution to the total CH loss rate is direct formation of H + CS + CO *via* pathway P3b, accounting for 22% of the CH loss rate at 150 K and increasing to 98% at 1000 K. Furthermore, the branching fractions predicted by MESMER for each individual reaction pathway indicate that the high energy pathways (P1a and P1b) forming H + CO + CS make no contribution to the total CH loss rate below 1600 K, and only P1b accounts for 0.5% of the overall reaction rate coefficient at 3000 K. This is a consequence of the significant barriers to formation of P1INT1 and P1INT2, which inhibits the reaction progressing *via* those routes at almost all temperatures. We also observe that formation of H + CO + CS *via* the lower energy pathway P1c only has a significant contribution above 1000 K, with the branching fraction increasing from 0.4% at 1000 K to 10.1% at 3000 K.

Figure S2 shows that at a certain temperature, the branching fractions for reaction products produced by reaction pathways P2 and P3b are equal. The temperature at which this

occurs, or the ‘crossover point,’ varies as a function of the total density. For example, the crossover point is 708 K and 608 K for total densities of $10^{17}$ cm$^{-3}$ and $10^{20}$ cm$^{-3}$, respectively. The trend here is that the crossover point occurs at a lower temperature for higher total densities. Our results presented in Figure S2 suggest CO + HCS should be recommended as the primary reaction products under conditions relevant to the ISM. We observe that the ‘crossover point’ occurs at a temperature of 1144 K for a total density of $10^{11}$ cm$^{-3}$, which reduces to 558 K at $10^{21}$ cm$^{-3}$. It is clear from our simulations that at low densities, the point at which the branching fractions of reaction pathways P2 and P3b are equal occurs at a much greater temperature than is typical for cold areas of the ISM (<100 K), providing further evidence in contradiction of the recommendations of the KIDA database.

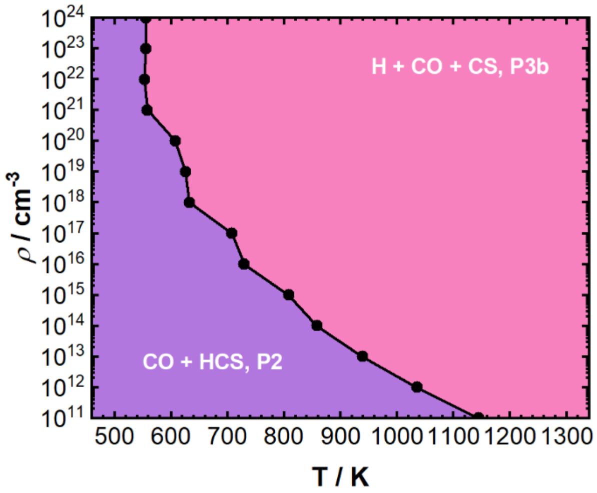


**Figure S2.** Variation of the temperature, K, for which the branching fraction of reaction pathway P2 is equal to P3b as a function of total density, cm$^{-3}$.

## MESMER Simulation with Experimental Data Fitting

A useful feature within the MESMER software package is the ability to utilise data fitting procedures to alter, for example, the simulated ILT parameters to result in *k(T)* fit to experimentally measured *k(T)*. It should be noted that the results from fitting implicitly accounts for redissociation. Data fitting procedures use a Marquardt least-squares fitting algorithm to minimise $\chi^2$ given by eq 2 to optimise parameters affecting the rate of reaction, namely the ILT parameters $A^\infty$ and $n^\infty$ which control the rate of association of two species in a barrierless unimolecular reaction.

$$\chi^2 = \sum \frac{\left(k_{experiment} - k_{calculated}\right)^2}{\sigma k_{experiment}{}^2} \tag{2}$$

Here, $k_{\text{experiment}}$, $k_{\text{calculated}}$ and $\sigma$ refers to the experimental reaction rate coefficient at a given temperature, the corresponding temperature dependent reaction rate coefficient calculated by MESMER for the same temperature and total density as the experimental value, and the experimental error in the value of the rate coefficient, respectively. This procedure was adopted here, with the results given in Table S11 (“Optimised Parameters”) and shown in Figure S3.

**Table S11.** Summary of input conditions for MESMER fitting exercise and optimised ILT parameters.

| **Initial Parameters** | | **Optimised Parameters** | | **$k(T)$ / $10^{-10}$ cm$^3$ s$^{-1}$** | | |
|---|---|---|---|---|---|---|
| **$A^\infty$ / $10^{-10}$ cm$^3$ s$^{-1}$** | **$n^\infty$** | **$A^\infty$ / $10^{-10}$ cm$^3$ s$^{-1}$** | **$n^\infty$** | **T / K** | **Experimental** | **MESMER** |
| 1.00 | -0.40 | 4.08 ± 0.10 | -0.03 ± 0.06 | *297* | *3.57 ± 0.25*[a] | -- |
| | | | | 297 | 3.90 ± 0.30 | 3.72 |
| | | | | 382 | 3.17 ± 0.15 | 3.41 |
| | | | | 472 | 2.85 ± 0.21 | 3.09 |
| | | | | 572 | 2.74 ± 0.07 | 2.76 |
| | | | | 667 | 2.69 ± 0.09 | 2.47 |

[a]This experimental data point determined at 30 Torr, while all other experimental measurements were performed at 100 Torr, and therefore this data point was not included in the fitting procedure.

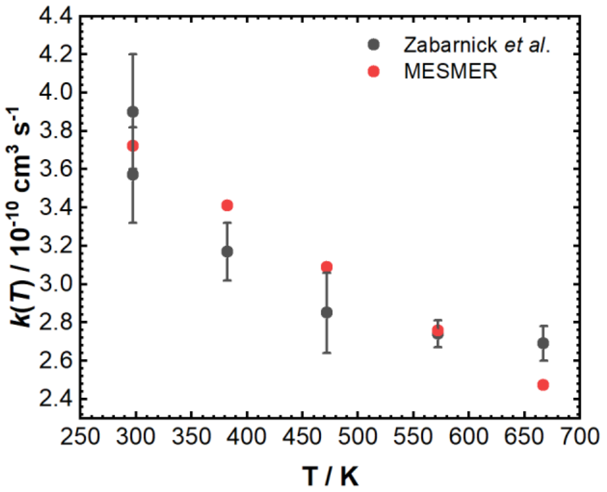


**Figure S3.** Calculated reaction rate coefficients from fitting the reaction PES model to available experimental data from Zabarnick *et al.*[5]

A value of 2.51 was achieved for $\chi^2/N$, a statistical measure of the goodness of fit. $\chi^2$ is given by eq 2 and $N$ is the number of degrees of freedom, which is the number of experimental data points, 5, minus the number of floated parameters, 1. The closer the ratio of $\chi^2/N$ is to 1, the better the fit. A higher value may suggest that the experimental error on the value of the rate coefficient is unrealistic, or that only altering the ILT parameters is not enough to capture the experimental data. Despite this higher value of $\chi^2/N$, the calculated value of the reaction rate coefficient is within 10% of the experimental values. However, the curved nature of the experimental data is not quite replicated in the MESMER calculated values based on our *ab initio* potential energy surface and the fitted ILT parameters. This is highlighted in Figure S3 and is not unsurprising considering that there are only five experimental data points for the CH + OCS reaction over a fairly small temperature range. Since such few experimental data points exist, it is difficult to know the high temperature and low temperature trend in $k(T)$, and how that affects the fitted parameters. Further optimisation is necessary, but would require additional experimental data over a wider range of temperatures in order to yield more accurate fitting parameters.

## Prior Distribution Analysis for Dissociation of HCS.

To examine the dissociation of HCS in further detail within MESMER, a Prior distribution methodology was initialised. The Prior distribution is a statistical model in which all degrees of freedom of the CH-OCS complex where CH inserts into the C–S bond of OCS, P2INT2, are treated equally with full randomisation of energy. Since CO is an initial product upon dissociation of the complex, CO departs with a distribution of internal and translational energies which then impacts the distribution of internal and translation energies that the HCS

molecule emerges with from the reaction. We can then look at how the nascent statistical distribution of HCS energies changes as a function of the initial energy going into the HCS + CO products, as the energy of P2INT2 is increased above the reaction threshold, displayed in Figure S4. The total fraction of HCS molecules that statistically have enough energy to decompose into H + CS can then provide an upper limit to the product branching fraction of decomposed HCS. From our *ab initio* calculations, the reaction threshold for formation of CO + HCS from P2INT2 is 39.5 kJ mol$^{-1}$ above the CO + HCS energy and $\Delta E_{CO+HCS\rightarrow H+CS+CO}$ = +208.0 kJ mol$^{-1}$, as highlighted in Figure S4. Therefore, the minimum internal energy of HCS above the reaction threshold required for HCS to undergo decomposition is 168.5 kJ mol$^{-1}$, if we consider the energy randomization in HCS through ergodicity would eventually lead to decomposition.

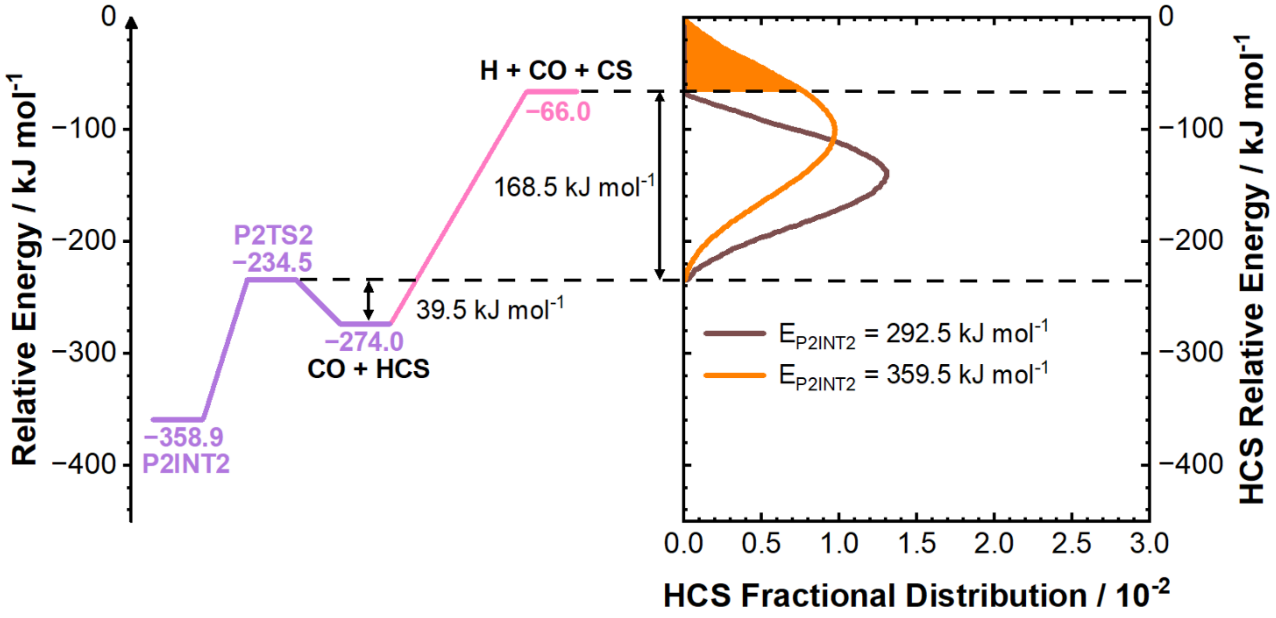


**Figure S4.** Model of the prior distribution analysis used to examine HCS decomposition. Energy values are quoted in kJ mol$^{-1}$ and are ZPVE corrected and scaled relative to the entrance channel. Left: Portion of the reaction PES for which this analysis was caried out. Right: The prior distribution of HCS for complex energies ($E_{P2INT2}$) of 292.5 (brown) and 359.5 (orange) kJ mol$^{-1}$, respectively, above the ZPVE correct electronic energy of the complex (-358.9 kJ mol$^{-1}$).

In Figure S4, two prior distributions of HCS are presented for P2INT2 complex energies of 292.5 (brown) and 359.5 (orange) kJ mol$^{-1}$, respectively, above the ZPVE correct electronic energy of the complex (-358.9 kJ mol$^{-1}$). If the complex energy is below the energy of H + CO + CS, then no HCS decomposition is expected from the statistical model as there is not enough internal energy. For the distribution shown in brown in Figure S4, the prior distribution model predicts that 0% of the HCS formed in the reaction can decompose to

H + CS. By considering P2INT2 having all 359.5 kJ $mol^{-1}$ available to it as being statistically distributed over its internal degrees of freedom, then the nascent statistical Prior distribution model predicts an upper limit to the total branching fraction of H + CO + CS of 0.19 (orange distribution). The more sophisticated MESMER simulated branching fraction of H + CS + CO at 150 K and $10^{17}$ $cm^{-3}$ is 0.22. Both the Prior distribution and the branching fractions calculated from phenomenological rate coefficients by MESMER below 800 K suggest that the primary products from the CH + OCS reaction are CO + HCS. Our results are consistent with KIDA above 800 K as H + CS + CO are expected to be the sole products from the title reaction.

## References


(1) Chase, M. *NIST-JANAF Thermochemical Tables*; American Institute of Physics, 1998.

(2) Butler, J. J.; Baer, T. Thermochemistry and Dissociation Dynamics of State-Selected $C_4H_4X$ Ions. 1. Thiophene. *Journal of the American Chemical Society* **1980**, *102* (22), 6764-6769. DOI: 10.1021/ja00542a016.

(3) Butler, J. J.; Baer, T.; Evans, S. A. Energetics and Structures of Organosulfur Ions: $CH_3SSCH_3^+$, $CH_3SS^+$, $C_2H_5S^+$, and $CH_2SH^+$. *Journal of the American Chemical Society* **1983**, *105* (11), 3451-3455. DOI: 10.1021/ja00349a012.

(4) Osborn, D. L.; Mordaunt, D. H.; Choi, H.; Bise, R. T.; Neumark, D. M.; Rohlfing, C. M. Photodissociation Spectroscopy and Dynamics of the HCCO Free Radical. *Journal of Chemical Physics* **1997**, *106* (24), 10087-10098. DOI: 10.1063/1.474064.

(5) Zabarnick, S.; Fleming, J. W.; Lin, M. C. Kinetics of CH Radical Reactions with $N_2O$, $SO_2$, OCS, $CS_2$, and $SF_6$. *International Journal of Chemical Kinetics* **1989**, *21* (9), 765-774. DOI: 10.1002/kin.550210905.
